# Evolution and Survival on Eutherian Sex Chromosomes

**DOI:** 10.1371/journal.pgen.1000568

**Published:** 2009-07-17

**Authors:** Melissa A. Wilson, Kateryna D. Makova

**Affiliations:** 1 Department of Biology, Pennsylvania State University, University Park, Pennsylvania, United States of America; 2 Center for Comparative Genomics and Bioinformatics, Pennsylvania State University, University Park, Pennsylvania, United States of America; 3 The Integrative Biosciences Program, Pennsylvania State University, University Park, Pennsylvania, United States of America; National Institute of Genetics, Japan

## Abstract

Since the two eutherian sex chromosomes diverged from an ancestral autosomal pair, the X has remained relatively gene-rich, while the Y has lost most of its genes through the accumulation of deleterious mutations in nonrecombining regions. Presently, it is unclear what is distinctive about genes that remain on the Y chromosome, when the sex chromosomes acquired their unique evolutionary rates, and whether X-Y gene divergence paralleled that of paralogs located on autosomes. To tackle these questions, here we juxtaposed the evolution of X and Y homologous genes (gametologs) in eutherian mammals with their autosomal orthologs in marsupial and monotreme mammals. We discovered that genes on the X and Y acquired distinct evolutionary rates immediately following the suppression of recombination between the two sex chromosomes. The Y-linked genes evolved at higher rates, while the X-linked genes maintained the lower evolutionary rates of the ancestral autosomal genes. These distinct rates have been maintained throughout the evolution of X and Y. Specifically, in humans, most X gametologs and, curiously, also most Y gametologs evolved under stronger purifying selection than similarly aged autosomal paralogs. Finally, after evaluating the current experimental data from the literature, we concluded that unique mRNA/protein expression patterns and functions acquired by Y (versus X) gametologs likely contributed to their retention. Our results also suggest that either the boundary between sex chromosome strata 3 and 4 should be shifted or that stratum 3 should be divided into two strata.

## Author Summary

Using recently available marsupial and monotreme genomes, we investigated nascent sex chromosome evolution in mammals. We show that, in eutherian mammals, X and Y genes acquired distinct evolutionary rates and functional constraints immediately after recombination suppression; X-linked genes maintained lower, ancestral (autosomal), rates, whereas the evolutionary rates of Y-linked genes increased. Most X and, unexpectedly, Y genes evolved under stronger purifying selection than similarly aged autosomal paralogs. However, we also observed that the divergence of gametologs and paralogs shared similar features. In addition, many Y-linked copies evolved unique functions and expression patterns compared to their counterparts on the X chromosome. Therefore, our results suggest that to be retained on the Y chromosome, genes need to acquire separately valuable expression and/or functions to be safeguarded by purifying selection.

## Introduction

Therian sex chromosomes, X and Y, evolved from a pair of homologous autosomes and thus originally harbored an identical set of genes [1]–[3]. Driven by a male-determining locus (*SRY*), the stepwise suppression of recombination between the Y and the X led to evolutionary strata corresponding to individual suppression events [1]. Suppression of recombination between the Y and the X also resulted in their current dramatically different gene numbers [2], ∼1,100 and <200 genes on the human X and Y, respectively [4],[5]. While many X-linked genes have been preserved, the majority of Y-linked genes have been pseudogenized or deleted. Purifying selection is predicted to be inefficient in nonrecombining regions of the Y, causing an accumulation of deleterious mutations; eventually, genes are expected to be lost by means of Muller's ratchet, background selection, the Hill-Robertson effect, and/or genetic hitchhiking of beneficial mutations [6],[7]. The already gene-poor mammalian Y continues to deteriorate [8], and it has been proposed that within a few million years the human Y will lose all of its genes, with major consequences for mankind [2],[9].

The human Y has retained a meager 16 functional single-copy protein-coding genes described as X-degenerate [10], i.e. possessing divergent X chromosome gametologs (*gametologs* are X-Y homologs [11]). Therefore, these genes represent relics of ancient autosomal genes (the remaining functional Y-linked genes are classified as pseudoautosomal, ampliconic, and recently X-transposed [5]). What evolutionary forces have been maintaining these X-degenerate genes on the Y? The first possibility is that the surviving genes might carry out essential functions where purifying selection maintains the amino acid sequence of the encoded protein leading to a low rate ratio of nonsynonymous to synonymous substitutions (K_A_/K_S_). However, decreased efficacy of such selection on the Y would elevate K_A_/K_S_ for Y vs. X gametologs [8]. The second possibility is that recombination suppression between the X and the Y can be viewed, effectively, as a duplication event. There are several proposed scenarios for how paralogs diverge from one another, including asymmetric evolution, where one copy is presumed to maintain the ancestral function, and thus experiences stronger purifying selection, while the other copy can undergo neofunctionalization or pseudogenization [12] and thus might experience positive selection or evolve neutrally. If this scenario holds true with respect to X and Y divergence, we expect that X gametologs will maintain the ancestral somatic functions necessary to both males and females (because the X is present in both sexes), and will evolve under purifying selection. Purifying selection might be strong on the X because it is hemizygous in males and thus recessive alleles are readily available for such selection to operate there. Y-linked genes, present only in males may undergo neofunctionalization, or, as has often been observed, may undergo pseudogenization [4],[5],[10]. Purifying selection is expected to be weak for genes on the Y because of the lack of recombination there (see above). Thus, similar to paralogs, divergence in function and expression between Y- and X-gametologs might actually contribute to the survival, in addition to the accelerated evolution [13], of Y chromosome genes.

Previous studies have observed elevated evolutionary rates for Y- versus X-linked genes. For instance, evolutionary rates were found to be higher for human and mouse Y chromosome genes compared with their gametologs on the X [13]. However, without available outgroup sequences, the incipient stages of X- and Y-linked gene evolution remained ambiguous, i.e., the ancestral sex chromosome branch could not be broken into X- and Y-specific segments. In a different study, not only was purifying selection shown to be less potent in exons of three primate Y than X chromosome genes, but positive selection was also evident at several sites of Y chromosome exons [8]. Nevertheless, as both sex chromosomes carry genes with a nonrandom assortment of functions (e.g., genes involved in spermatogenesis are enriched on the Y [14], whereas genes important for reproduction and brain function are overrepresented on the X [2]), contrasting only the X- and Y-linked genes might not represent an ideal way to study the evolution of either gene group. When feasible, a direct comparison of sex chromosome genes with homologous autosomal genes is therefore warranted.

Tied to the understanding of sex chromosome evolution are hypotheses of how X and Y diverged from each other forming different evolutionary strata. Each stratum corresponds to a distinct recombination suppression event, thus, gametologs belonging to the same stratum have similar divergence [1]. In eutherian mammals, five strata of increasing age are observed linearly along the X chromosome, with the youngest near its proximal end and the oldest near its distal end, suggesting that suppression of recombination occurred in a stepwise manner between X and Y [1],[4]. The arrangement of homologous sequences on the Y chromosome has been scrambled, supporting the hypothesis about the role of inversions in Y chromosome evolution [1],[4].

While some X-degenerate Y chromosome genes were retained from the original autosomal pair, others were added later. After eutherian-marsupial divergence (∼166 MYA [15]), the eutherian sex chromosomes acquired the X-/Y-added region (XAR/YAR), through a translocation from an autosome [16]. This segment remains autosomal in marsupials and monotremes [16],[17] and provides a direct comparison of homologous genes between autosomes and sex chromosomes. Such a comparison allows us to infer the eutherian proto-sex chromosome branch and separate the ancestral sex chromosome branch into X- and Y-specific portions, i.e. to investigate emergent eutherian sex chromosome evolution.

In eutherian mammals, the XAR/YAR continued to recombine between X and Y until the formation of strata 3 and 4, app roximately 80–130 MYA and 30–50 MYA, respectively [1]. Primates and rodents diverged ∼85–90 MYA [18], and thus genes belonging to stratum 3 putatively began evolving as X- and Y-specific in the ancestor of eutherian mammals. It is expected that stratum 4 genes only evolved as X- and Y-specific along the primate lineage. Only 12 human gametologous pairs with functional Y homologs are left in the human XAR/YAR [1],[4]: TMSB4X/Y, CX/YORF15A, CX/YORF15B, EIF1AX/Y, ZFX/Y, USP9X/Y, DDX3X/Y, and UTX/Y are classified in stratum 3 [1],[4]; but there has been some debate whether stratum 4 contains PRKX/Y, NLGN4X/Y, TBL1X/Y, and AMELX/Y (classified based on sequence divergence [1]) or whether TBL1X/Y and AMELXY/Y belong, instead, to stratum 3 (based on analysis of parsimonious inversions [4]).

Here, in our attempt to analyze the early stages of sex chromosome evolution, as well as to address what evolutionary forces lead to preservation of functional Y chromosomal gametologs, we analyzed 12 XAR/YAR gametologous pairs in eutherians along with their autosomal orthologs in opossum and platypus.A direct comparison of homologs decreased biases due to sequence composition, gene size, and ancestral functional constraints possible in studies juxtaposing Y- and X-linked genes against nonhomologous autosomal genes (e.g., [19]). Specifically, we tested the following hypotheses: 1) whether X and Y evolved unique evolutionary rates immediately after the suppression of recombination between them; 2) whether the evolutionary rates along both the X and Y branches have been constant throughout their evolutionary histories, and, 3) whether gametolog evolution parallels paralog evolution in terms of rates and functional constraints. Additionally, by utilizing whole-genome transcriptome and other published experimental data, we examined whether the expression and functional divergence of Y from X gametologs correlated with their evolution and potentially contributed to their survival on the sex chromosomes. Because of the use of opossum and platypus sequences, for the first time we are able to get a glimpse of how the ancestral eutherian sex chromosomes evolved.

## Results/Discussion

### Pre- and post-radiation tree topologies

**Figure 1:**
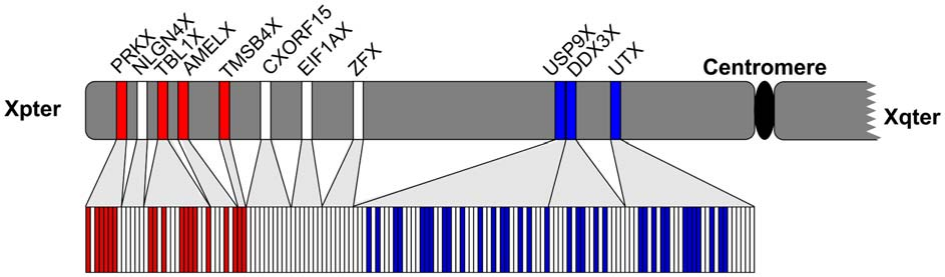
Phylogenetic analysis and branch length comparisons for concatenated gene sequences: gene-by-gene (upper panel) and exon-by-exon (lower panel) analysis. Xpter and Xqter—the termini of the short and long arms of the X chromosome, respectively. Red and blue boxes indicate the post- and pre-radiation topology, respectively, and white boxes represent masked out sequence (see Materials and Methods).

**Figure 2:**
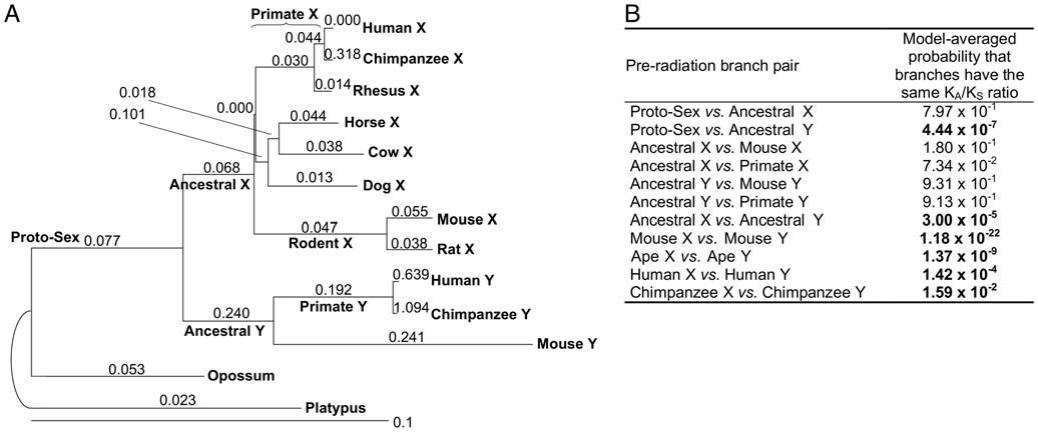
Pre-radiation phylogeny and evolutionary rate comparisons. (A) Phylogeny for the pre-radiation topology. Exons with less than 50% bootstrap support for clades with either the pre- or post-radiation topology, fewer than 24 nucleotides aligned across all species, or inconsistent with the topology of the whole gene were excluded. Branch lengths are proportional to the estimated synonymous substitutions per site, and are labeled with the nonsynonymous-to-synonymous rate ratios (K_A_/K_S_). (B) Branch length comparisons for the pre-radiation topology. We present the model-averaged probabilities (not *P* values) that two branches have the same Ka/Ks ratio, and so corrections for multiple tests are neither needed nor appropriate (see Materials and Methods). Significant values are shown in bold.

**Figure 3:**
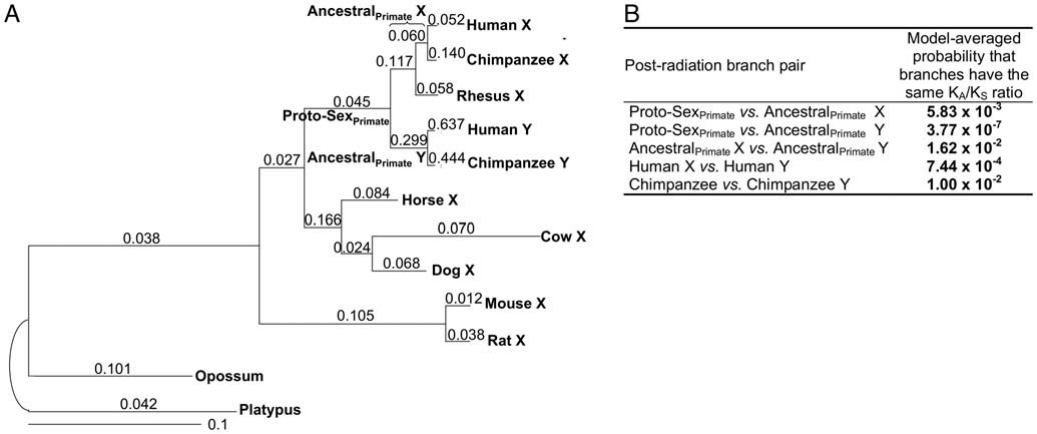
Post-radiation phylogeny and evolutionary rate comparisons. (A) Phylogeny for the post-radiation topology. Exons with less than 50% bootstrap support for clades with either the pre- or post-radiation topology, fewer than 24 nucleotides aligned across all species, or inconsistent with the topology of the whole gene were excluded. Branch lengths are proportional to the estimated synonymous substitutions per site, and are labeled with the nonsynonymous-to-synonymous rate ratios (K_A_/K_S_). (B) Branch length comparisons for the post-radiation topology. We present the model-averaged probabilities (not *P* values) that two branches have the same Ka/Ks ratio, and so corrections for multiple tests are neither needed nor appropriate (see Materials and Methods). Significant values are shown in bold.

To test the hypotheses stated above, we studied the evolution of all 12 available XAR/YAR human functional gametologs [4]: PRKX/Y, NLGN4X/Y, TBL1X/Y, AMELX/Y, TMSB4X/Y, CX/YORF15A, CX/YORF15B, EIF1AX/Y, ZFX/Y, USP9X/Y, DDX3X/Y, and UTX/Y, here listed starting from the Xpter (Figure 1; the Y-linked gametolog of CXorf15 in human and chimpanzee has been split into two genes, CYorf15A and CYorf15B [10], which we investigate separately). We included sequences from eight eutherian mammals (human, chimpanzee, rhesus, horse, cow, dog, mouse and rat) that had sufficient sequence coverage for robust analysis of all of the genes in the XAR (Figure 2, Figure 3, and Materials and Methods) as well as human, chimpanzee and (when available) mouse YAR gene sequences. To isolate chromosome-specific effects and to delineate the ancestral and proto-sex chromosomes branches, we included the orthologous autosomal gene sequences from opossum and platypus. In opossum, the order of genes found in the XAR/YAR is the same as in eutherians, but the sequences are split between chromosomes 4 and 7 [20]. The platypus genome is not yet assembled, however, the presence of the orthologous genes on a single chicken chromosome (chromosome 1) [4], in the same order, suggests that the original translocation likely occurred in one event.

The phylogenetic analysis of the coding region within each homologous XAR/YAR gene group usually resulted in one of two separate tree topologies. For DDX3X/Y, USP9X/Y, and UTX/Y, we observed the *pre-radiation tree topology* (Figure 1, Figure 2, Figure S1), in which X- and Y-linked genes formed two distinct clades, and thus these gametologs diverged from one another in the common ancestor of boreoeutherian mammals [21], forming stratum 3, believed to be shared among all eutherian mammals [1]. For PRKX/Y, NLGN4X/Y, TBL1X/Y, AMELX/Y, and TMSB4X/Y, we observed the *post-radiation tree topology* (Figure 1, Figure 3, Figure S1), in which primate gametologs clustered together, and therefore recombination suppression between them followed the boreoeutherian radiation and presumably occurred along the primate lineage, forming stratum 4. For genes with the post-radiation topology, consistent with previous experimental assays [22]–[24], we did not identify the homologous mouse Y genes, suggesting that they have been deleted, pseudogenized beyond the recognition of the alignment algorithms utilized, or are yet unsequenced (Materials and Methods). For each gene with either the pre- or post-radiation topology, the observed topology was significantly different from the alternative topology (Table S1). Genes for which the topology could not be confidently determined, CX/Yorf15A, CX/Yorf15B, EIF1AX/Y and ZFX/Y (Figure S1), were excluded from the concatenated analysis (Table S1), along with NLGN4X/Y (Figure S1), because its murid X orthologs could not be identified reliably [25].

**Figure 4:**
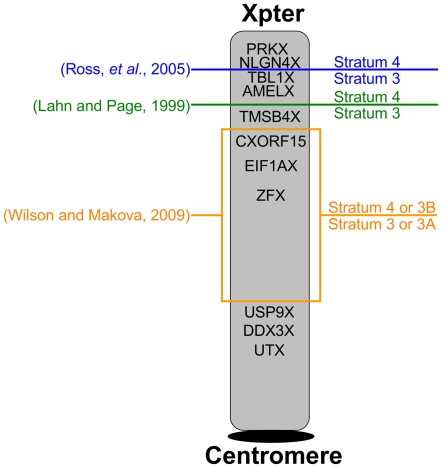
New stratum boundary. The previous descriptions of the stratum3–stratum4 boundary are shown, along with a new boundary region, identified by this study.

To test for gene conversion, we conducted a phylogenetic analysis of each exon individually. Exons where the X and Y sequence from the same species formed a unique clade have putatively undergone gene conversion and were excluded from further analysis (Table S2). In most cases though, the phylogenetic trees produced for each exon were identical to the topology of the parent gene. When exons following the post- and pre-radiation topology were mapped to the X chromosome, they grouped closest and furthest from the Xpter, respectively (Figure 1) in a significantly non-random distribution (P<2.2×10^−16^; Wilcoxon rank-sum test). Although gene conversion was detected for isolated exons (Table S2), the observed distribution is more parsimoniously explained by two distinct evolutionary strata. Thus, either the boundary separating strata 3 and 4, is closer to the position suggested in [1], i.e. between TMSB4X and AMELX, or it is located between TBL1X and NLGN4X, as proposed in [4], but stratum 3 should be split into two sub-strata with a second boundary somewhere between USP9X and TMSB4X (Figure 4).

### Comparison of evolution among X, Y, and autosomal genes

Homologous marsupial and monotreme sequences have allowed us to expand upon previous efforts investigating sex chromosome evolution [13]. In particular, for the pre-radiation topology, we were able to separate the ancestral sex chromosome branch (preceding the boreoeutherian divergence) into X- and Y-specific portions (labeled Ancestral X and Ancestral Y, respectively, Figure 2A) and to delineate the eutherian proto-sex chromosome branch (labeled Proto-Sex, Figure 2A), preceding the Y chromosome inversion that led to formation of stratum 3. Similarly, for primates in the post-radiation topology, we were able to investigate the evolution of X- and Y-linked sequences before (identified by the Proto-Sex_Primate_ branch) and after the recombination suppression event that led to the formation of stratum 4 (indicated on the Ancestral_Primate_X and Ancestral_Primate_Y branches).

To study differences in evolutionary rates of X, Y, and autosomal genes, we concatenated the coding regions of genes following the pre-radiation (PRKX/Y, TBL1X/Y, AMELX/Y and TMSB4X/Y; a total of 2700 bp) and post-radiation (USP9X/Y, DDX3X/Y and UTX/Y; a total of 6108 bp) topology separately (Materials and Methods, Table S1; bootstrap values shown in Figure S2), to reduce the confounding influences of comparing genes from potentially different strata. Further, we masked out exons from the exon-by-exon analysis described above that (1) did not conform to the topology characteristic for the majority of the exons of the gene (these are likely gene conversion events), (2) produced an ambiguous tree topology, or (3) lacked sufficient data (see Materials and Methods).

First, we investigated how synonymous rates differ among the two sex chromosomes and the homologous autosomal sequence. Synonymous rates for genes with the pre-radiation topology (Figure 2) were significantly higher for Y than X gametologs (between the sum of branches to the common ancestor between human X and Y, *P* = 1.01×10^−3^; chimpanzee X and Y, *P* = 1.31×10^−3^; and mouse X and Y, *P* = 4.40×10^−6^), reflecting male mutation bias [26]. Genes with this topology had significantly higher synonymous rates for mouse than human (compared between the sum of branches to the common ancestor, *P* = 2.43×10^−10^ for mouse X - human X, *P* = 2.54×10^−10^ for mouse Y - human Y), in agreement with previous studies (e.g., [27]). Synonymous rates for genes with the post-radiation topology (Figure 2B) were (not significantly) higher between mouse X vs. human X, and similar between human Y and X sums of branches (data not shown).

Synonymous rates were lower in the opossum lineage (0.282 and 0.530 for the pre- and post-radiation topology, respectively) than in even the shortest eutherian lineages (0.469 and 1.227; calculated as the sum of eutherian-specific branches leading to Human X for the pre-radiation topology and Horse X for the post-radiation topology, respectively). This can be explained by the lower GC content and reduced recombination rates of opossum vs. eutherian chromosomes [20],[28]. The differences in opossum rates between the pre- and post-radiation topologies might either result from interchromosomal rate variation [29], since most of the genes following the former and latter topologies are found on opossum chromosomes 4 and 7, respectively, or be driven by local genomic factors [30].

Second, we studied variation in the K_A_/K_S_ ratio among branches. For every comparison in both topologies, the K_A_/K_S_ ratio was significantly higher for the Y than the X branch (Figure 2B, Figure 3B). Our data set allowed us to investigate when these differences between X and Y chromosome evolution emerged, i.e. whether the elevated evolutionary rates observed on the Y versus the X occurred immediately after recombination suppression or just recently, after million years of suppressed recombination. For both topologies, immediately after recombination suppression, the Y chromosome (Ancestral Y and Ancestral_primate_ Y branches for pre- and post-radiation, respectively) acquired significantly higher K_A_/K_S_ ratios as compared with the Proto-Sex branch (Figure 2B, Figure 3B). This increase could be due to relaxed purifying selection on the Y in the absence of recombination and/or due to positive selection of Y-linked genes that acquired new functions [8]. Positive selection was not detected on any branches or sites in these seven genes (see Materials and Methods) and, consequently, K_A_/K_S_ ratios were interpreted as varying degrees of purifying selection, reflecting the level of functional constraints. Thus, purifying selection was weaker on the Ancestral Y branch than on the Proto-Sex branch (or the Ancestral X branch) for trees with both topologies (Figure 2B, Figure 3B). In contrast, the intensity of purifying selection did not differ significantly between the Proto-Sex and Ancestral X branches for gametologs following the pre-radiation topology, implying that these X-linked genes have retained the level of functional constraints of their autosomal ancestors (Figure 2B).

Interestingly, X and Y lineages of the pre-radiation topology maintained relatively constant K_A_/K_S_ ratios since the suppression of recombination between them (Figure 2B; recent gametolog separation in the post-radiation topology prevented us from conducting a similar analysis there). Indeed, the K_A_/K_S_ ratio was not significantly different between the Ancestral X branch and either the ape or the mouse X branches, again suggesting preservation of functional constraints of X gametologs. Similarly, the K_A_/K_S_ ratio did not differ significantly between the Ancestral Y branch and either the ape or the mouse Y branches, indicating that Y rapidly settled on its own equilibrium evolutionary rate [13].

### Comparing evolution of gametologs and autosomal paralogs

We next asked whether divergence between gametologs mimicked the divergence between paralogs. To answer this question, we compared the evolution of human gametologs (here all 12 gametologous pairs were considered) against pairs of similarly aged human autosomal paralogs. Using the synonymous rate (K_S_) as an estimate of evolutionary age, for each gametolog, we compiled a set of similarly aged autosomal trios composed of a pair of human paralogs, duplicated after human-opossum divergence, aligned with the orthologous autosomal sequence in opossum (a total of 470 trios; Materials and Methods). The distribution of pairwise K_A_/K_S_ ratios was significantly different between gametologs and similarly aged autosomal paralogs (*P* = 0.0001, Wilcoxon test). The impact of positive selection was minor (only 13 sites of CYorf15B and 5 sites of ZFY exhibited signatures of positive selection; Materials and Methods), and thus we again interpreted the K_A_/K_S_ ratio as the strength of purifying selection. Pairwise K_A_/K_S_ ratios were lower for nine out of 12 gametologs than for autosomal paralogs (Table 1), suggesting stronger purifying selection acting on gametologs. The higher pairwise K_A_/K_S_ ratios observed for AMELX/Y, CX/Yorf15A and CX/Yorf15B might reflect the initial stages of Y gametolog pseudogenization [10],[31] or positive selection acting on some CYorf15B sites. Stronger purifying selection between most gametologs than paralogs contradicts the hypothesis of sexual selection driving more rapid divergence between gametologs than autosomal paralogs [32].

**Table 1:** Contrasting the evolution of gametologs and autosomal paralogs.

	Pairwise				Branch-specific								
Gametologs	X vs. Y				X copy vs. slow paralog				Y copy vs. fast paralog				Asymmetry
	K_A_/K_S_ a	#^b^	Med^c^	*P* d	K_A_/K_S_ a	#^b^	Med^c^	*P* d	K_A_/K_S_ a	#^b^	Med^c^	*P* d	*GAbranch* e
PRKX/Y	0.276	66	0.531	*0.197*	0.000	356	1.036	***0.000***	0.466	111	1.574	*0.324*	*0.387*
NLGN4X/Y	0.128	219	0.500	*0.205*	0.028	360	0.000	*0.572*	0.164	407	8.341	*0.123*	*0.880*
TBL1X/Y	0.172	41	0.527	*0.049*	0.106	44	0.289	*0.136*	0.165	75	0.423	*0.080*	*0.984*
AMELX/Y	1.265	206	0.474	*0.864*	0.096	365	0.703	*0.584*	3.267	381	1.734	*0.811*	*0.378*
TMSB4X/Y	0.156	49	0.525	*0.082*	0.000	348	0.000	***0.000***	0.119	36	0.348	*0.111*	*0.706*
CX/Yorf15A	0.505	38	0.477	*0.553*	0.000	46	0.299	*0.283*	0.746	43	0.586	***0.000***	*0.871*
CX/Yorf15B	0.654	39	0.480	*0.872*	0.332	51	0.151	*0.471*	0.557	47	0.380	*0.529*	*0.643*
EIF1AX/Y	0.006	34	0.392	*0.029*	0.000	58	0.274	***0.000***	0.015	51	0.533	***0.000***	*0.521*
ZFX/Y	0.175	62	0.536	*0.097*	0.048	109	0.186	*0.284*	0.162	64	0.657	*0.063*	*0.640*
USP9X/Y	0.100	32	0.445	*0.094*	0.031	44	0.289	*0.091*	0.125	37	0.339	*0.135*	***0.000***
DDX3X/Y	0.077	38	0.447	*0.053*	0.006	60	0.298	*0.050*	0.130	40	0.411	*0.125*	***0.001***
UTX/Y	0.250	34	0.502	*0.118*	0.093	108	0.401	*0.315*	0.256	38	0.508	*0.184*	*0.128*

Gametologs were compared against similarly aged paralogs. Age was approximated by the rate of synonymous substitutions (K_S_); empirical distributions of K_S_ for the autosomal paralogs, determined individually for each gametolog or gametolog pair were composed of all autosomal paralogs with a K_S_ value within ±0.1 of the branch-specific or pairwise K_S_, respectively.

a the nonsynonymous-to-synonymous rate ratio.

b the number of similarly aged paralogs (see Materials and Methods).

c median K_A_/K_S_ ratio for the similarly aged paralogs.

d the one-tailed empirical P value for the significance in difference between a value for gametologs and the median value for paralogs. P values shown in bold were significant after Bonferroni correction for multiple tests.

e the model-averaged probability of K_A_/K_S_ ratios being equal between the X and Y copies (see Materials and Methods).

Using opossum sequence to polarize substitutions, we determined that most gametologs displayed asymmetric functional constraints, meaning that the K_A_/K_S_ ratios differed between the two gametologs, often by an order of magnitude, although not always significantly so, and all gametologs had a lower K_A_/K_S_ ratio for the X than Y copy (Table 1). Thus, gametologs likely followed an evolutionary scenario proposed for paralogs, in which purifying selection was stronger for one than the other paralogous copy [12]. And, consistent with our expectation (see Introduction), purifying selection was always stronger for the X than the Y copy.

We next asked whether X and Y gametologs evolved at rates similar to these for slowly and quickly evolving paralogous copies, respectively (slowly and quickly evolving paralogous copies were determined using opossum as an outgroup). In contrast to expectations of inefficient purifying selection on the Y [6], all but three Y gametologs had lower K_A_/K_S_ ratios and thus may have evolved under stronger purifying selection than the quickly evolving copies of paralogs (Table 1). This might represent a mechanism of Y gametolog preservation; either a gene must be maintained through purifying selection, or, as evident again for AMELY, CYorf15A, and CYorf15B, that had higher K_A_/K_S_ ratios than the similarly aged quickly evolving paralogs, they may become prey to pseudogenization. Relatively strong purifying selection observed for Y gametologs might also, in part, be explained by genetic hitchhiking due to selection acting on other Y chromosome genes (e.g., ampliconic genes); genetic hitchhiking is expected to be particularly potent on the Y because it does not undergo recombination outside of the pseudoautosomal regions.

Similar to Y gametologs, all but two X gametologs had lower K_A_/K_S_ ratios than the slowly evolving paralogous copies (Table 1). Intense purifying selection acting on X gametologs can be explained by the fact that X is hemizygous in males (thus recessive alleles are instantly open to selection) and by the preservation of somatic functions important for both sexes.

### Divergence in gene expression and function between gametologs

**Figure 5:**
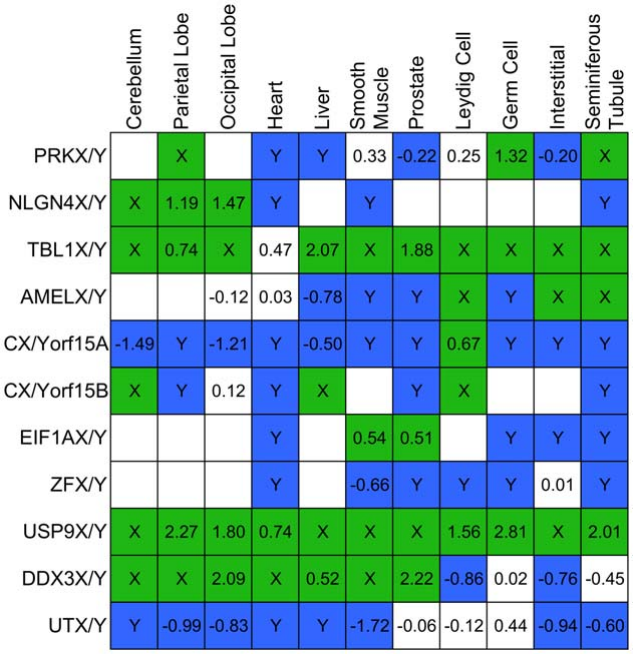
Tissue-specific divergence between human X and Y gametologs. We compared divergence in gene expression based on the presence or absence of gametolog expression and, when both gametologs in a pair were expressed, used the fold change to compare the expression levels between the two gametologs in each pair (see Materials and Methods). Blue field indicates tissues in which the Y gametolog is expressed at a higher level than the X gametolog; green field indicates tissues in which the X gametolog is expressed at a higher level than the Y gametolog; white field with a value indicates similar (less than 25% different) expression for X and Y; and an empty white field indicates that neither gametolog is expressed in a particular tissue. Numbers represent log_2_(*X*/*Y*), where *X* and *Y* are X and Y expression values, respectively. Labels “X” or ”Y” indicate that only the X or only the Y gametolog is expressed. The data for all 11 gametologous pairs present on the array from a study by Su and colleagues [33] are shown (TMSB4X/Y pair was not present on the array).

To test a hypothesis that the expression and functional divergence of Y gametologs from their X counterparts potentially contributed to the survival of the former on the sex chromosomes, we compiled and analyzed whole-genome transcriptome and other published experimental data. Expression divergence between X and Y gametologs was inferred from human and mouse transcriptome microarray data produced by Su and colleagues [33]. In humans we studied 11 tissue samples collected from males in that study. In over three quarters of gametolog-tissue combinations, either the X and Y gametologs in a pair were expressed at unequal levels (at least 25% different) or one copy was completely turned off (Figure 5). Thus, gametologs acquired expression patterns distinct from one another.

We found no significant difference in the expression divergence between human gametologous pairs and similarly aged human autosomal paralogs (Table S3), implying that the expression patterns of gametologous pairs diverge from one another at a similar rate as those of paralogous pairs. Next, using the proportion of tissues in which both the X and Y gametolog are similarly expressed (white boxes with a number in Figure 5) among all tissues with detected expression as a measure of gametolog expression similarity, we determined that there is no significant difference in expression patterns between gametologs following the pre- versus post-radiation topologies (Wilcoxon rank sum test, *P* = 0.3018), and there is no significant correlation (*P* = 0.622) between gametolog expression similarity and the distance from the Xpter. The non-significance may be due to both the limited number of genes, as well as the limited number of tissues available for the analysis. However, given that expression patterns diverge very rapidly, frequently outpacing sequence divergence [34],[35], the genes considered here may already have diverged past any threshold of observing certain correlations.

Mouse samples used in the study of Su and colleagues [33], were all pooled from tissues collected from both males and females, thus it was impossible to distinguish levels of X and Y expression unambiguously. Still, two mouse Y-linked genes included in microarrays analyzed by Su and colleagues [33] - Ddx3y and Usp9y - had undetectable expression across all 61 tissues analyzed, while their gametologs, Ddx3x and Usp9x were expressed in all and one of the tissues examined, respectively (the other gametologs present on the array studied, Utx/y and Zfx/y, were not expressed [33]). Therefore, we do observe unique expression patterns between at least some mouse and most human X and Y gametologs. These differences in expression might have contributed to the retention of Y gametologs.

Additionally, mining and compiling nearly 15 years of experimental data gathered from the literature allowed us to conclude that the majority of human X and Y gametologs acquired unique protein expression patterns and/or functions (Table S4), sometimes not detectable from studies of gene expression alone. For instance, in the case of human DDX3X/Y, although both gametologs are widely transcribed, only the X-linked copy, DDX3X, is also widely translated, while DDX3Y is translated exclusively in the male germ line [36]. This is accompanied by distinct temporal protein expression patterns, at least in spermatogenesis, where the two protein products are present at different stages [36]. In another example, the TBL1X/Y gametologs differ in both mRNA expression and protein function. TBL1X mRNA is ubiquitously expressed [37], while TBL1Y mRNA expression is limited to only a few tissues [38]. The dissimilarity is also evident in function as the TBL1X protein represses transcription [39], while the TBL1Y protein has no such activity [38]. As a final example, AMELY deletions cause no detectable phenotypic changes [40], but deletion of AMELX causes amelogenesis imperfecta [31],[41]. Such differences in protein expression and function between gametologs might have also contributed to retention of X degenerate Y chromosome genes.

### Conclusion

To the best of our knowledge, we present the first analysis of the ancestral proto-sex evolutionary rates in eutherian mammals. We observed that immediately following the suppression of recombination between X and Y, likely due to their importance in both sexes, X gametologs largely maintained the ancestral autosomal sequence and functional constraints. In contrast, Y gametologs, as predicted due to absence of recombination [6], evolved under weaker purifying selection than X gametologs. Further, these different rates have been roughly maintained through evolutionary time by each of the sex chromosomes. Both X and Y gametologs, on average, acquired functional constraints stronger than quickly and slowly evolving copies of autosomal paralogs, respectively. This might have contributed to the survival of these gametologs. We also observe that the divergence between of X and Y gametolog sequences after recombination suppression, in some ways mimics that of paralogous genes, were one copy maintains a lower, more conservative, rate of evolution while the other is allowed a higher substitution rate, and may eventually evolve a new function or become prey to pseudogenization. Our analysis of the sequence evolution combined with experimental observations suggests that to withstand the evolutionary vulnerability on the Y chromosome, most Y-linked genes diverged in expression and function from their X gametologs to become separately valuable.

Although Y chromosome sequencing and assembly is an undeniably challenging endeavor [5],[10],[42], it provides invaluable and otherwise impossible insights into mammalian evolution. Further studies investigating gametologs will critically depend on the availability of Y chromosome sequences for several mammals, in addition to human [5] and chimpanzee [42].

## Materials and Methods

### Sequence collection

Eutherian X-linked and corresponding autosomal nucleotide sequences for opossum and platypus were extracted from the 28-way vertebrate alignments [43] available through Galaxy [44], using the human X homolog as a reference. We initially considered X-linked sequences from all 18 eutherian species included in the 28-way genomic alignments [43], but retained only eight due to limited coverage in the other species (Figure 2 and Figure 3). Only complete human and chimpanzee Y [5],[10], and partial mouse Y chromosome sequences are available. Human, chimpanzee and mouse Y-linked sequences were downloaded from Genbank (see Table S5). Of the 12 gametologs, we identified only four (Zfy, Usp9y, Ddx3y, and Uty) annotated on the mouse Y chromosome in Genbank. Since the mouse Y chromosome has yet to be completely sequenced and assembled, we searched the available 533 mouse Y BACs (a total of ∼90 Mb) for the seven missing genes. Using BlastZ [45], we identified the four previously annotated genes (see above), but were unable to locate the unannotated genes.

### Phylogenetic analysis and tests for gene conversion

The coding nucleotide sequences for each homologous gene group (sex-linked gametologs and autosomal homologs) were aligned using ClustalW [46]. The phylogenetic trees were built according to the Neighbor-Joining method [47] as implemented in PHYLIP [48] using X-linked sequences from human, chimpanzee, rhesus, mouse, rat, cow, dog, horse, Y-linked sequences from human, chimpanzee, and mouse, when available, and autosomal sequences from opossum and platypus. These species were chosen among the 18 mammals represented in [43] because for each of them at least nine of the 12 genes had greater than 50% sequence coverage. 1000 bootstrap replicates were generated first for each gene and then for each coding exon. Exons with less than 50% bootstrap support for clades with either the pre- or post-radiation topology, fewer than 24 nucleotides aligned across all species, or inconsistent with the topology of the whole gene (a total of 92 exons) were excluded from this portion of the analysis. In addition to Neighbor-Joining analysis, we used Maximum Likelihood and Maximum Parsimony tree building methods [48]. The three approaches led to similar results (data not shown). Our results represent gene trees, not necessarily species trees (see discussion of primate, rodent, and carnivore groupings in [49]), and so we advise against using these groupings to support arguments for or against contentious species groupings.

The exon by exon analysis described above led us to identify known cases of gene conversion (e.g. in ZFX/Y [50]). To further test for gene conversion, we aligned human X with human Y, chimp X with chimp Y and mouse X with mouse Y sequences using PipMaker [51], a software that utilizes a local alignment algorithm to output regions of similar sequence identity. Higher identity of a particular stretch of an alignment in relation to the entire alignment can be suggestive of gene conversion [52]. New instances of gene conversion were not detected either with this method nor with GENECONV [53].

### Synonymous/nonsynonymous rates and tests for positive selection

HyPhy was used to estimate the branch-specific K_S_ and K_A_ under the GY94_3×4 model and to test for statistical significance of differences in the synonymous rates among branches using a Likelihood Ratio Test (LRT), testing the likelihood that two branches had the same vs. different K_S_ values [54]. Tests conducted with the MG94_3×4 and MG94xHKY_3×4 models yielded similar statistically significant results. To compute the probability that the K_A_/K_S_ ratio was significantly different between two branches, we used the GAbranch analysis [55] in the online version of HyPhy (www.datamonkey.org), which computes the model-averaged probability that two branches have the same K_A_/K_S_ ratio [56]. To determine the significance of the difference between sums of branches, we re-ran our analyses excluding the species that broke the branches we intended to compare (e.g., in the pre-radiation topology, we excluded rat X to be able to compare mouse X and Y branches). To examine a possibility of positive selection, we first used the GAbranch analysis [55],[56] to compute the model-averaged probability that K_A_ was significantly greater than K_S_ along each branch. Second, we tested for significant differences between site-specific models M1 (neutral) and M2 (selection), and between M7 (beta) and M8 (beta and omega >1) in the codeml module of PAML [57]. Selection was not detected by these two methods. In a third test for positive selection, using the random effects likelihood (REL) approach [56],[58] to identify specific sites that might have been acted on by positive selection, there was evidence for positive selection at 13 sites of CYorf15B and at 5 sites of ZFY.

### Comparison with autosomal paralog evolution

Using the FASTA method [59], 6,536 autosomal paralogous pairs were identified among 48,218 protein sequences of consensus CDS, known, and novel genes in Ensembl (release 38 of NCBI build 36). Each human protein in a paralogous pair was used as a blastp query against all known opossum proteins [45]. An opossum homolog was identified if it was the highest scoring hit to both human paralogs with an e-value <1×10^−10^. A pair of human paralogs together with the opossum homolog formed a trio that was retained if, after computing branch-specific K_A_ and K_S_ in the codeml module of PAML [57], K_S_ was <1 along the sum of the two human branches, to ensure that the human paralogs were duplicated after human-opossum divergence [20]. Finally, gene trios were excluded if any of the three genes were sex-linked in their respective species, or if the absolute value of the difference between the K_A_/K_S_ ratios of human paralogs, Δ(K_A_/K_S_), was greater than 10. As a result, a total of 470 trios were retained.

Pairwise K_A_ and K_S_ were estimated for each gametologous pair (without masking any exons) and for each paralogous pair, using the codeml module of PAML [57]. Using the opossum homolog as an outgroup to polarize the changes, we then identified the slowly and quickly evolving copies for each gametologous or paralogous gene pair as the gene having a lower or higher K_A_/K_S_ ratio relative to each other, respectively. The K_A_/K_S_ ratio for each X-linked gametolog was compared against the distribution of these ratios calculated for the slowly evolving paralogous gene copies, and the K_A_/K_S_ ratio for each Y gametolog was compared against the distribution of these ratios calculated for the fast evolving paralogous gene copies. We computed the probability that the observed pairwise or branch-specific K_A_/K_S_ ratio for each gametolog was significantly lower than these values calculated for paralogs by calculating a left-tailed empirical P value, equal to the number of paralogs having a lower ratio than a gametologous pair under consideration, divided by the total number of paralogs. Empirical distributions for the autosomal paralogs, determined individually for each gametolog, were composed of all autosomal paralogs with a K_S_ value within ±0.1 of the pairwise or branch-specific K_S_ of the gametolog(s). The significance of the results did not change if we used a range of ±0.05, and only changed for one pair if we used a range of ±0.5. Final P values were corrected for multiple comparisons according to the Bonferroni method. The probability that the X- and Y-specific branches for each gametologous pair had significantly different K_A_/K_S_ ratios was estimated using the GAbranch analysis [55] implemented in the online version of HyPhy [56].

### Expression analysis

To analyze human and mouse gametologous gene expression, we used the data from [33]. Probe sets were mapped to genes and screened for potential cross-hybridization to both gametologs in each pair following the methods described in [60]. Reliable probe sets were identified for all human and mouse gametologous pairs (Table S6). For humans, all but 13 of the 79 tissues analyzed in [33] were either female-specific or pooled between females and males. Of the remaining 13, we used only 11 that were non-redundant tissues [33]. For a gene to be considered expressed in a particular tissue, we required the average difference (AD) to be greater than 200 in that tissue, following a method described by Su and colleagues [33]. If both genes in a pair were expressed, we calculated the fold change, F_k_, computed as the log of the ratio of X and Y expression, log_2_(X/Y). If the Y-linked gene is more highly expressed than its X gametolog, F_k_ will be negative, whereas if the X gametolog is more highly expressed, F_k_ will be positive. For −0.25<F_k_<0.25, we considered X and Y to be similarly expressed. The results did not change qualitatively if we used a larger range of −0.5<F_k_<0.5.

Distributions of autosomal paralogs were taken from the pairwise analysis, described above (so that we compare the expression divergence of each gametologous pair with similarly aged autosomal paralogs, as measured by K_S_). Reliable probe sets and expression values were identified following the methods described above. Empirical P values were computed as explained for paralogs.

### Functional differentiation

Gametolog functional and protein expression data were retrieved from the iHOP (Information Hyperlinked Over Proteins) database (http://www.ihop-net.org/UniPub/iHOP/), the OMIM (Online Mendelian Inheritance of Man) database (http://www.ncbi.nlm.nih.gov/omim/), and PubMed (http://www.ncbi.nlm.nih.gov/PubMed/).

## Supporting Information

Figure S1 Gene-specific synonymous trees built according to the Neighbor-Joining method. The complete coding sequence for each gene is evaluated. Bootstrap support from 1,000 replicates is indicated as a percentage along each branch.
(0.37 MB DOC)

Figure S2 Bootstrap values for concatenated trees. (A) Pre-radiation topology with bootstrap values. The concatenated coding sequence for the genes in the pre-radiation topology are evaluated (USP9X/Y, DDX3X/Y and UTX/Y). (B) Post-radiation topology with bootstrap values. The concatenated coding sequence for the genes in the pre-radiation topology are evaluated (PRKX/Y, TBL1X/Y, AMELX/Y and TMSB4X/Y). Bootstrap support from 1,000 replicates is indicated as a percentage along each branch.
(0.07 MB DOC)

Table S1 The numbers of base pairs analyzed for each gene. The numbers of base pairs per (human) gene, excluded and analyzed for either the pre- or post-radiation topology. P indicates the P value from the Kishino-Hasegawa test [1] comparing whether the observed topology (pre- or post-radiation) is significantly different from the alternative topology (post- or pre-radiation). Unresolved topologies were compared against both pre- and post-radiation topologies. Genes are listed in the order of increasing distance from the Xpter.
(0.07 MB DOC)

Table S2 Exon by exon phylogenetic analysis. X's indicate less than 50% sequence coverage in a given species. The other mammalian species not shown in the table (armadillo, bushbaby, cat, elephant, guinea pig, hedgehog, rabbit, shrew, tenrec, and treeshrew) were excluded completely. The set of 12 orthologous XAR genes was assessed in each species to determine the percentage of alignable nucleotides (sequence coverage), relative to the human X-linked sequences. Species were excluded if fewer than nine of the 12 XAR genes had less than 50% sequence coverage. For AMELX/Y, additional Y-linked sequences were included in the phylogenetic analysis because their complete coding sequences were available in GenBank from previous studies. No other complete YAR gametolog sequences were available in GenBank at the time of this study.
(0.62 MB DOC)

Table S3 Comparison of gametolog versus autosomal paralog expression. Expression divergence, measured as the number of tissues out of 11 in which the genes are differentially expressed (see Materials and Methods) is compared for each gametolog pair. X vs. Y represents the number of tissues in which X and Y are differentially expressed, Paralog represents the median number of tissues in which the similarly aged (see Materials and Methods) autosomal paralogs are differentially expressed, and P represents the empirical P value indicating whether the gametolog pair is significantly more differentially expressed than similarly aged autosomal paralogous pairs.
(0.05 MB DOC)

Table S4 Functional differences between the studied XAR/YAR gametologs. The unique functions reported for either the X copy or the Y copy are listed in each respective column. Functions similar for both the X and the Y copy are listed across both columns.
(0.15 MB DOC)

Table S5 Accession numbers for all complete YAR genes, retrieved from GenBank. Listed are the NCBI accession numbers for all available complete coding sequences of orthologous Y-linked genes in mammals, at the time of this study.
(0.06 MB DOC)

Table S6 Identification of optimal probe sets. To identify gene-specific probe sets, we used the consensus sequence for each probe set as a query in blastn [1] against the nonreduntant human (A) or mouse (B) genomes. Database hits were considered from known proteins with an e-value less than or equal to 1×10^−20^ and either (1) an identity of 100% and length greater than 49 bp, or (2) an identity higher than 94% and length of at least either 99 bp or 90% of the length of the query. If more than two specific probes were identified, we used the longest one.
(0.09 MB DOC)
