# Supplementary material for: Evolution and Survival on Eutherian Sex Chromosomes
Source: PLoS Genet. 2009 Jul 17;5(7):e1000568. doi: 10.1371/journal.pgen.1000568 (PMC2704370; doi:10.1371/journal.pgen.1000568)

**Figure S1.** **Gene-specific synonymous trees built according to the Neighbor-Joining method.** The complete coding sequence for each gene is evaluated. Bootstrap support from 1000 replicates is indicated as a percentage along each branch.

1. USP9X/Y


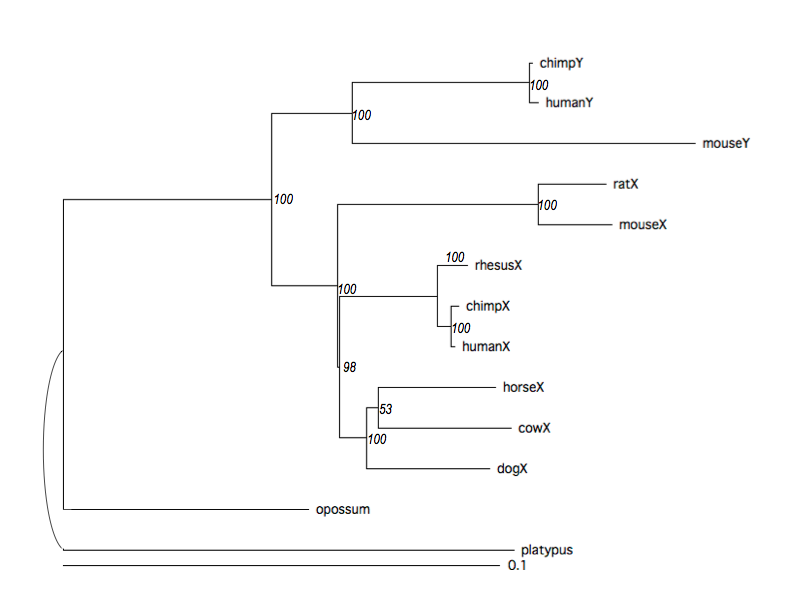


1. DDX3X/Y


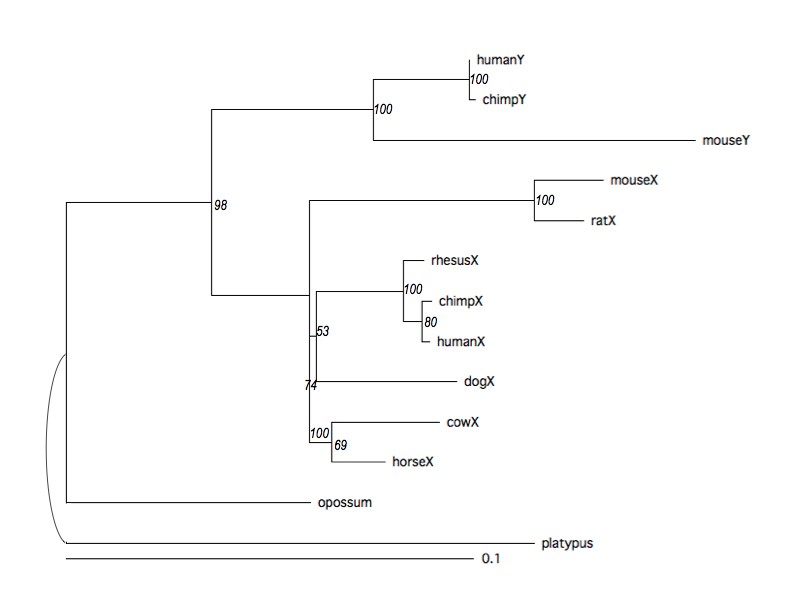


1. UTX/Y


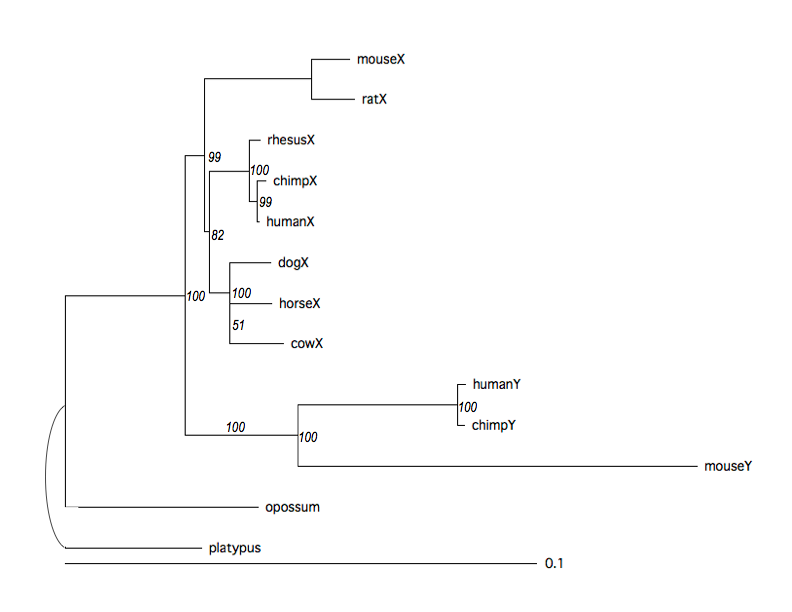


1. PRKX/Y


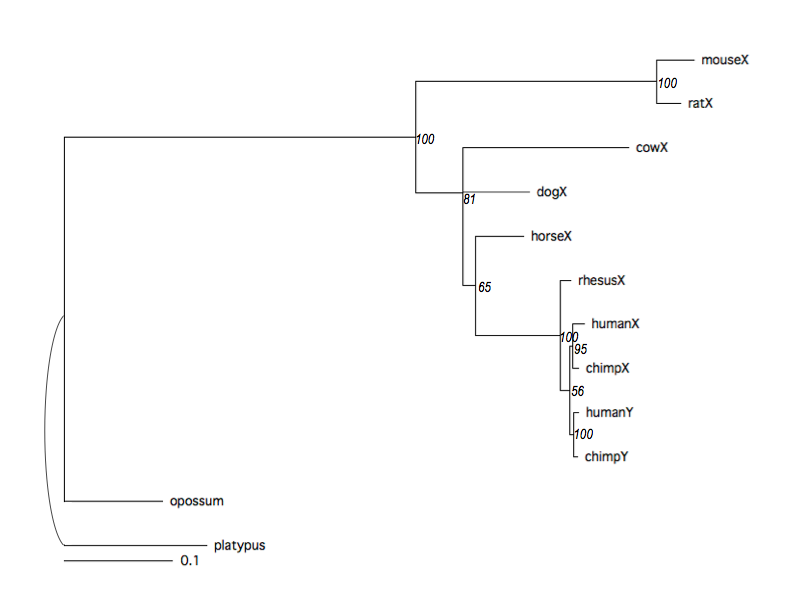


1. NLGN4X/Y


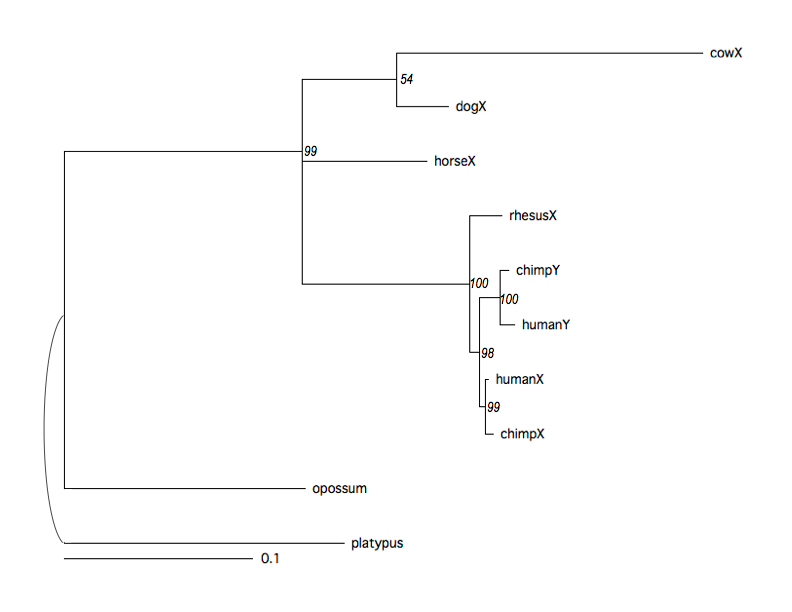


1. TBL1X/Y


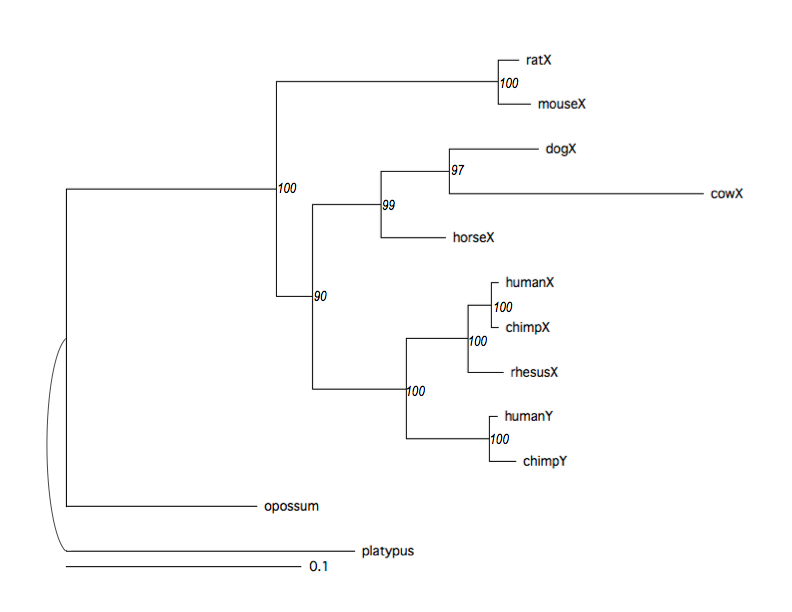


1. AMELX/Y


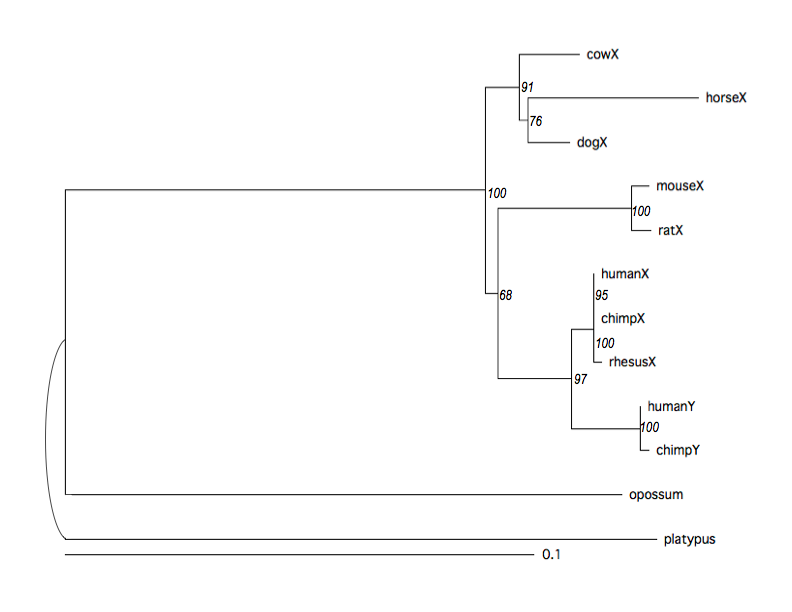


1. TMSB4X/Y


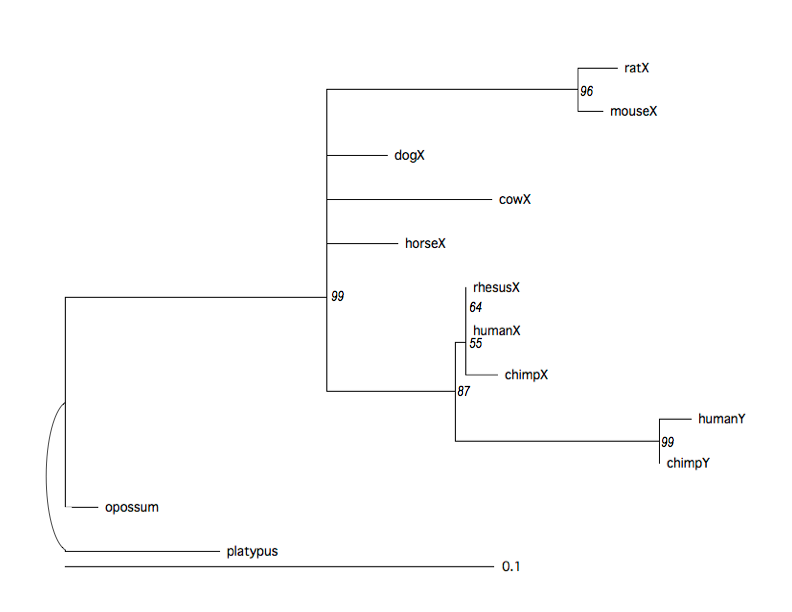


1. CX/Yorf15A


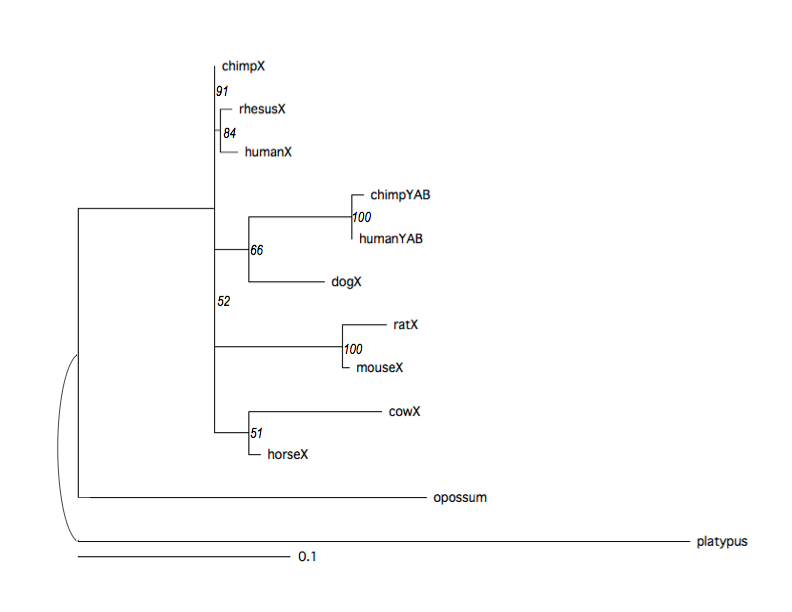


1. CX/Yorf15B


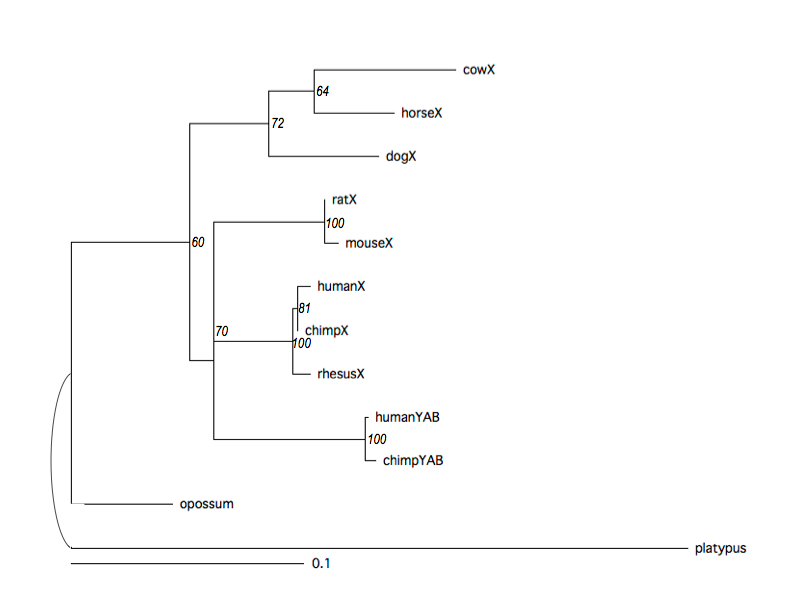


1. EIF1AX/Y


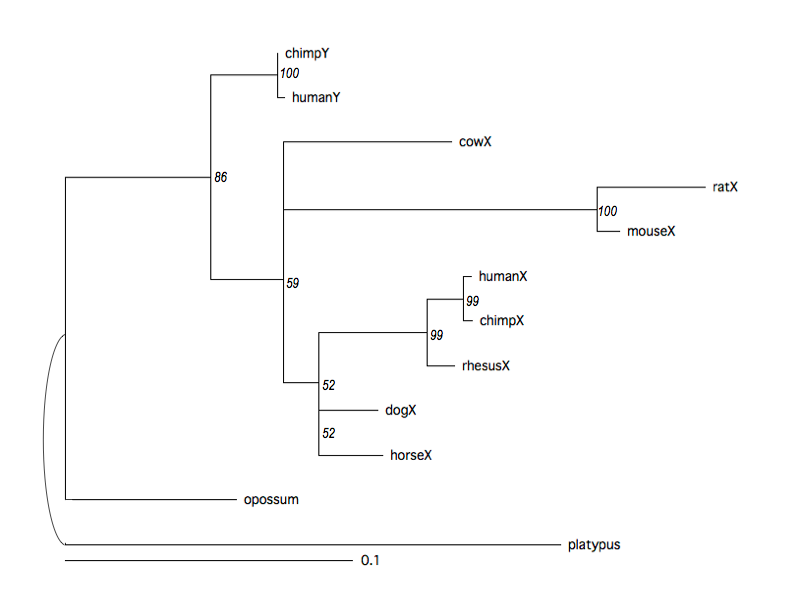


1. ZFX/Y


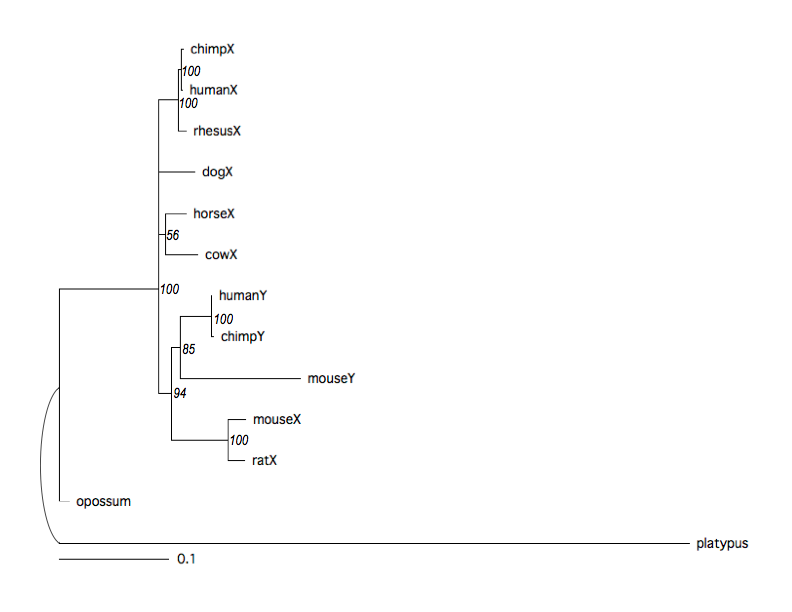

Supplement: Figure S1 — Gene-specific synonymous trees built according to the Neighbor-Joining method. The complete coding sequence for each gene is evaluated. Bootstrap support from 1,000 replicates is indicated as a percentage along each branch. (0.37 MB DOC) [file pgen.1000568.s001.doc]
