# Supplementary material for: Evolution and Survival on Eutherian Sex Chromosomes
Source: PLoS Genet. 2009 Jul 17;5(7):e1000568. doi: 10.1371/journal.pgen.1000568 (PMC2704370; doi:10.1371/journal.pgen.1000568)

**Figure S2.** **Bootstrap values for concatenated trees. A. Pre-radiation topology with bootstrap values.** The concatenated coding sequence for the genes in the pre-radiation topology are evaluated (USP9X/Y, DDX3X/Y and UTX/Y). **B. Post-radiation topology with bootstrap values.** The concatenated coding sequence for the genes in the pre-radiation topology are evaluated (PRKX/Y, TBL1X/Y, AMELX/Y and TMSB4X/Y). Bootstrap support from 1000 replicates is indicated as a percentage along each branch.


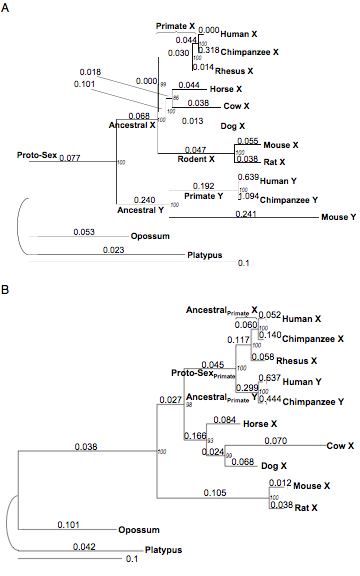

Supplement: Figure S2 — Bootstrap values for concatenated trees. (A) Pre-radiation topology with bootstrap values. The concatenated coding sequence for the genes in the pre-radiation topology are evaluated (USP9X/Y, DDX3X/Y and UTX/Y). (B) Post-radiation topology with bootstrap values. The concatenated coding sequence for the genes in the pre-radiation topology are evaluated (PRKX/Y, TBL1X/Y, AMELX/Y and TMSB4X/Y). Bootstrap support from 1,000 replicates is indicated as a percentage along each branch. (0.07 MB DOC) [file pgen.1000568.s002.doc]
