# Supplementary material for: Evolution and Survival on Eutherian Sex Chromosomes
Source: PLoS Genet. 2009 Jul 17;5(7):e1000568. doi: 10.1371/journal.pgen.1000568 (PMC2704370; doi:10.1371/journal.pgen.1000568)
Supplement: Table S1 — The numbers of base pairs analyzed for each gene. The numbers of base pairs per (human) gene, excluded and analyzed for either the pre- or post-radiation topology. P indicates the P value from the Kishino-Hasegawa test [1] comparing whether the observed topology (pre- or post-radiation) is significantly different from the alternative topology (post- or pre-radiation). Unresolved topologies were compared against both pre- and post-radiation topologies. Genes are listed in the order of increasing distance from the Xpter. (0.07 MB DOC) [file pgen.1000568.s003.doc]

**Table S1. The numbers of base pairs analyzed for each gene.** The numbers of base pairs per (human) gene, excluded and analyzed for either the pre- or post-radiation topology. P indicates the P value from the Kishino-Hasegawa test [1] comparing whether the observed topology (pre- or post-radiation) is significantly different from the alternative topology (post- or pre-radiation). Unresolved topologies were compared against both pre- and post-radiation topologies. Genes are listed in the order of increasing distance from the Xpter.

| Gametologs | Whole Gene Topology | P | Total  Base Pairs | Base Pairs Excluded | Base Pairs Analyzed |
| --- | --- | --- | --- | --- | --- |
| PRKX/Y | Post-radiation | 0 | 1077 | 126 | 951 |
| NLGN4X/Y | Post-radiation | 0 | 2451 | 2451 | 0 |
| TBL1X/Y | Post-radiation | 0 | 1734 | 597 | 1137 |
| AMELX/Y | Post-radiation | 0 | 618 | 138 | 480 |
| TMSB4X/Y | Post-radiation | 0 | 132 | 0 | 132 |
| Total for this topology | Post-radiation |  | 6012 | 3312 | 2700 |
| CX/Yorf15A | Unresolved | 0/0 | 396 | 396 | 0 |
| CX/Yorf15B | Unresolved | 0/0 | 546 | 546 | 0 |
| EIF1AX/Y | Unresolved | 0/0 | 432 | 432 | 0 |
| ZFX/Y | Unresolved | 0/0 | 2472 | 2472 | 0 |
| Total for this topology | Unresolved |  | 4497 | 4497 | 0 |
| USP9X/Y | Pre-radiation | 0 | 7740 | 4382 | 3358 |
| DDX3X/Y | Pre-radiation | 0 | 2001 | 1293 | 708 |
| UTX/Y | Pre-radiation | 0 | 4218 | 2176 | 2042 |
| Total for this topology | Pre-radiation |  | 13959 | 7851 | 6108 |

1. Kishino, H.,Hasegawa, M. (1989) Evaluation of the maximum likelihood estimate of the evolutionary tree topologies from DNA sequence data, and the branching order in hominoide*a J Mol Ev*ol 29, 170-179.
