# Supplementary material for: Evolution and Survival on Eutherian Sex Chromosomes
Source: PLoS Genet. 2009 Jul 17;5(7):e1000568. doi: 10.1371/journal.pgen.1000568 (PMC2704370; doi:10.1371/journal.pgen.1000568)
Supplement: Table S2 — Exon by exon phylogenetic analysis. X's indicate less than 50% sequence coverage in a given species. The other mammalian species not shown in the table (armadillo, bushbaby, cat, elephant, guinea pig, hedgehog, rabbit, shrew, tenrec, and treeshrew) were excluded completely. The set of 12 orthologous XAR genes was assessed in each species to determine the percentage of alignable nucleotides (sequence coverage), relative to the human X-linked sequences. Species were excluded if fewer than nine of the 12 XAR genes had less than 50% sequence coverage. For AMELX/Y, additional Y-linked sequences were included in the phylogenetic analysis because their complete coding sequences were available in GenBank from previous studies. No other complete YAR gametolog sequences were available in GenBank at the time of this study. (0.62 MB DOC) [file pgen.1000568.s004.doc]

**Table S2. Exon by exon phylogenetic analysis.** X’s indicate less than 50% sequence coverage in a given species. The other mammalian species not shown in the table (armadillo, bushbaby, cat, elephant, guinea pig, hedgehog, rabbit, shrew, tenrec and treeshrew) were excluded completely. The set of 12 orthologous XAR genes was assessed in each species to determine the percentage of alignable nucleotides (sequence coverage), relative to the human X-linked sequences. Species were excluded if fewer than nine of the 12 XAR genes had less than 50% sequence coverage.  For AMELX/Y, additional Y-linked sequences were included in the phylogenetic analysis because their complete coding sequences were available in GenBank from previous studies. No other complete YAR gametolog sequences were available in GenBank at the time of this study.

| Gene_Exon | Exon Length  (bp) | chimp X | chimp Y | cow X | cow Y | dog X | horse X | horse Y | human X | human Y | mouse X | mouse Y | opossum | pig Y | platypus | rat X | rhesus X | Description |
| --- | --- | --- | --- | --- | --- | --- | --- | --- | --- | --- | --- | --- | --- | --- | --- | --- | --- | --- |
| PRKX_1 | 126 | x | x |  |  |  |  |  | x | x | x |  |  |  | x | x | x | Unresolved |
| PRKX_2 | 78 | x | x |  |  |  |  |  | x | x | x |  |  |  | x | x | x | Post-radiation |
| PRKX_3 | 58 | x | x |  |  | x | x |  | x | x | x |  | x |  | x | x | x | Post-radiation |
| PRKX_4 | 96 | x | x |  |  | x | x |  | x | x | x |  | x |  | x | x | x | Post-radiation |
| PRKX_5 | 120 | x | x |  |  | x | x |  | x | x | x |  | x |  | x | x | x | Post-radiation |
| PRKX_6 | 264 | x | x |  |  | x | x |  | x | x | x |  | x |  | x | x | x | Post-radiation |
| PRKX_7 | 169 | x | x |  |  | x | x |  | x | x | x |  | x |  | x | x | x | Post-radiation |
| PRKX_8 | 166 | x | x |  |  | x | x |  | x |  | x |  | x |  | x | x | x | Post-radiation |
| NLGN4X_1 | 850 | x |  |  |  | x | x |  | x | x |  |  | x |  | x |  | x | Post-radiation |
| NLGN4X_2 | 790 | x | x | x |  | x | x |  | x | x |  |  | x |  | x |  | x | Post-radiation |
| NLGN4X_3 | 186 | x | x | x |  | x | x |  | x | x |  |  | x |  | x |  | x | Post-radiation |
| NLGN4X_4 | 153 | x | x | x |  | x | x |  | x | x |  |  | x |  | x |  | x | Post-radiation |
| NLGN4X_5 | 472 | x | x | x |  | x | x |  | x | x |  |  | x |  | x |  | x | Post-radiation |
| TBL1X_1 | 103 | x |  |  |  |  |  |  | x |  |  |  |  |  |  |  | x | No Y coverage |
| TBL1X_2 | 108 | x | x | x |  | x | x |  | x | x | x | x | x |  | x | x | x | Post-radiation |
| TBL1X_3 | 146 | x | x | x |  | x | x |  | x | x | x | x | x |  | x | x | x | Post-radiation |
| TBL1X_4 | 259 | x | x | x |  | x | x |  | x | x | x | x | x |  | x | x | x | Post-radiation |
| TBL1X_5 | 133 |  | x | x |  | x | x |  | x | x | x | x | x |  | x | x | x | Unresolved |
| TBL1X_6 | 142 | x | x | x |  | x | x |  | x | x | x | x | x |  | x | x | x | Post-radiation |
| TBL1X_7 | 64 | x | x | x |  | x | x |  | x | x | x | x | x |  | x | x | x | Unresolved |
| TBL1X_8 | 98 | x | x | x |  | x | x |  | x | x | x | x | x |  | x | x | x | Post-radiation |
| TBL1X_9 | 61 | x | x | x |  | x | x |  | x | x | x | x | x |  | x | x | x | Unresolved |
| TBL1X_10 | 122 | x | x | x |  | x | x |  | x | x | x | x |  |  | x | x | x | Post-radiation |
| TBL1X_11 | 75 | x | x | x |  | x | x |  | x | x | x | x |  |  | x | x | x | Post-radiation |
| TBL1X_12 | 128 | x | x | x |  | x | x |  | x | x | x | x |  |  | x | x | x | Post-radiation |
| TBL1X_13 | 166 | x | x | x |  | x | x |  | x | x | x | x |  |  | x | x | x | Post-radiation |
| TBL1X_14 | 102 | x | x | x |  | x | x |  | x | x | x | x |  |  | x | x | x | Unresolved |
| TBL1X_15 | 27 | x | x | x |  | x | x |  | x | x | x | x |  |  | x | x | x | Unresolved |
| AMELX_1 | 54 | x | x | x | x | x | x | x | x | x | x | x | x | x | x | x | x | Post-radiation |
| AMELX_2 | 48 | x | x | x | x | x | x | x | x | x | x | x | x | x | x | x | x | Unresolved |
| AMELX_3 | 42 | x |  | x |  | x | x |  | x |  | x |  |  | x |  | x | x | No Y coverage |
| AMELX_4 | 42 | x | x | x | x | x | x | x | x | x | x | x | x | x | x | x | x | Unresolved |
| AMELX_5 | 426 | x | x | x | x | x | x | x | x | x | x | x | x | x | x | x | x | Post-radiation |
| AMELX_6 | 6 | x | x | x | x | x | x | x | x | x | x | x | x |  | x | x | x | Unresolved |
| TMSB4X_1 | 100 | x | x | x |  | x | x |  | x | x | x | x | x |  | x | x | x | Post-radiation |
| TMSB4X_2 | 32 | x | x | x |  | x | x |  | x | x | x | x | x |  | x | x | x | Post-radiation |
| CXORF15_1 | 102 | x |  | x |  | x | x |  | x |  | x |  | x |  | x | x | x | No Y coverage |
| CXORF15_2 | 304 |  | x | x |  | x | x |  | x | x | x | x | x |  | x | x | x | Unresolved |
| CXORF15_3 | 92 | x | x | x |  | x | x |  | x | x | x | x | x |  | x | x | x | Unresolved |
| CXORF15_4 | 171 | x |  | x |  | x | x |  | x |  | x | x | x |  | x | x | x | No Y coverage |
| CXORF15_5 | 195 | x |  | x |  | x | x |  | x |  | x | x | x |  | x | x | x | No Y coverage |
| CXORF15_6 | 120 | x |  | x |  | x | x |  | x |  | x | x | x |  | x | x | x | No Y coverage |
| CXORF15_7 | 75 | x |  | x |  | x | x |  | x |  | x | x | x |  | x | x | x | No Y coverage |
| CXORF15_8 | 93 | x | x | x |  | x | x |  | x | x | x | x | x |  | x | x | x | Unresolved |
| CXORF15_9 | 96 | x | x | x |  | x | x |  | x | x | x | x | x |  | x | x | x | Unresolved |
| CXORF15_10 | 345 | x | x | x |  | x | x |  | x | x | x | x | x |  | x | x | x | Unresolved |
| EIF1AX_1 | 6 |  | x | x |  | x | x |  | x | x | x | x | x |  |  | x | x | Unresolved |
| EIF1AX_2 | 92 | x | x | x |  | x | x |  | x | x | x | x | x |  | x | x | x | Unresolved |
| EIF1AX_3 | 82 | x | x | x |  | x | x |  | x | x | x | x | x |  | x | x | x | Unresolved |
| EIF1AX_4 | 51 | x | x | x |  | x | x |  | x | x | x | x | x |  | x | x | x | Unresolved |
| EIF1AX_5 | 104 | x | x | x |  | x | x |  | x | x | x | x | x |  | x | x | x | Unresolved |
| EIF1AX_6 | 84 | x | x | x |  | x | x |  | x | x | x | x | x |  | x | x | x | Inconsistent: Pre |
| EIF1AX_7 | 13 | x | x | x |  | x | x |  | x | x | x | x | x |  | x | x | x | Unresolved |
| ZFX_1 | 61 | x | x | x |  | x | x |  | x | x | x | x | x |  | x | x | x | Inconsistent: Pre |
| ZFX_2 | 615 | x | x | x |  | x | x |  | x | x | x | x | x |  | x | x | x | Unresolved |
| ZFX_3 | 150 | x | x | x |  | x | x |  | x | x | x | x | x |  | x | x | x | Unresolved |
| ZFX_4 | 144 | x | x | x |  | x | x |  | x | x | x | x | x |  | x | x | x | Unresolved |
| ZFX_5 | 156 | x | x | x |  | x | x |  | x | x | x | x | x |  | x | x | x | Unresolved |
| ZFX_6 | 141 | x | x | x |  | x | x |  | x | x | x | x | x |  | x | x | x | Unresolved |
| ZFX_7 | 1205 | x | x | x |  | x | x |  | x | x | x | x | x |  | x | x | x | Gene Conversion |
| USP9X_1 | 96 | x | x | x |  | x | x |  | x | x | x | x | x |  | x | x | x | Unresolved |
| USP9X_2 | 149 | x | x | x |  | x | x |  | x | x | x | x | x |  | x | x | x | Unresolved |
| USP9X_3 | 80 | x | x | x |  | x | x |  | x | x | x | x | x |  | x | x | x | Unresolved |
| USP9X_4 | 113 |  | x | x |  | x | x |  | x | x | x | x | x |  | x | x | x | Pre-radiation |
| USP9X_5 | 219 | x | x | x |  | x | x |  | x | x | x | x | x |  | x | x | x | Unresolved |
| USP9X_6 | 116 | x | x | x |  | x | x |  | x | x | x | x | x |  | x | x | x | Pre-radiation |
| USP9X_7 | 252 | x | x | x |  | x | x |  | x | x | x | x | x |  | x | x | x | Unresolved |
| USP9X_8 | 139 | x | x | x |  | x | x |  | x | x | x | x | x |  | x | x | x | Unresolved |
| USP9X_9 | 153 | x | x | x |  | x | x |  | x | x | x | x | x |  | x | x | x | Unresolved |
| USP9X_10 | 105 | x | x | x |  | x | x |  | x | x | x | x | x |  | x | x | x | Pre-radiation |
| USP9X_11 | 207 | x | x | x |  | x | x |  | x | x | x | x | x |  | x | x | x | Pre-radiation |
| USP9X_12 | 137 | x | x | x |  | x | x |  | x | x | x | x | x |  | x | x | x | Unresolved |
| USP9X_13 | 134 | x | x | x |  | x | x |  | x | x | x | x | x |  | x | x | x | Unresolved |
| USP9X_14 | 88 | x | x | x |  | x | x |  | x | x | x | x | x |  | x | x | x | Unresolved |
| USP9X_15 | 343 | x | x | x |  | x | x |  | x | x | x | x | x |  | x | x | x | Unresolved |
| USP9X_16 | 96 | x | x | x |  | x | x |  | x | x | x | x | x |  | x | x | x | Pre-radiation |
| USP9X_17 | 215 | x | x | x |  | x | x |  | x | x | x | x | x |  | x | x | x | Pre-radiation |
| USP9X_18 | 241 | x | x |  |  | x | x |  | x | x | x | x | x |  | x | x | x | Pre-radiation |
| USP9X_19 | 153 | x | x | x |  | x | x |  | x | x | x | x | x |  | x | x |  | Unresolved |
| USP9X_20 | 121 | x | x | x |  | x | x |  | x | x | x | x | x |  | x | x | x | Unresolved |
| USP9X_21 | 131 | x | x | x |  | x | x |  | x | x | x | x | x |  | x | x | x | Pre-radiation |
| USP9X_22 | 279 | x | x | x |  | x | x |  | x | x | x | x | x |  | x | x | x | Pre-radiation |
| USP9X_23 | 126 | x | x | x |  | x | x |  | x | x | x | x | x |  | x | x | x | Unresolved |
| USP9X_24 | 126 | x | x | x |  | x | x |  | x | x | x | x | x |  | x | x | x | Unresolved |
| USP9X_25 | 170 | x | x | x |  | x | x |  | x | x | x | x | x |  | x | x | x | Pre-radiation |
| USP9X_26 | 109 | x | x | x |  | x | x |  | x | x | x | x | x |  | x | x | x | Unresolved |
| USP9X_27 | 147 | x | x | x |  | x | x |  | x | x | x | x | x |  | x | x | x | Unresolved |
| USP9X_28 | 147 | x | x | x |  | x | x |  | x | x | x | x | x |  | x | x | x | Unresolved |
| USP9X_29 | 223 | x | x | x |  | x | x |  | x | x | x | x | x |  | x | x | x | Pre-radiation |
| USP9X_30 | 221 | x | x | x |  | x | x |  | x | x | x | x | x |  | x | x | x | Unresolved |
| USP9X_31 | 191 | x | x | x |  | x | x |  | x | x | x | x | x |  | x | x | x | Unresolved |
| USP9X_32 | 174 | x | x | x |  | x | x |  | x | x | x | x | x |  | x | x | x | Pre-radiation |
| USP9X_33 | 142 | x | x | x |  | x | x |  | x | x | x | x | x |  | x | x | x | Unresolved |
| USP9X_34 | 754 | x | x | x |  | x | x |  | x | x | x | x | x |  | x | x | x | Pre-radiation |
| USP9X_35 | 124 | x | x | x |  | x | x |  | x | x | x | x | x |  | x | x | x | Pre-radiation |
| USP9X_36 | 238 | x | x | x |  | x | x |  | x | x | x | x | x |  | x | x | x | Unresolved |
| USP9X_37 | 130 | x | x | x |  | x | x |  | x | x | x | x | x |  | x | x | x | Unresolved |
| USP9X_38 | 186 | x | x | x |  | x | x |  | x | x | x | x | x |  | x | x | x | Pre-radiation |
| USP9X_39 | 221 | x | x | x |  | x | x |  | x | x | x | x | x |  | x | x | x | Unresolved |
| USP9X_40 | 89 | x | x | x |  | x | x |  | x | x | x | x | x |  | x | x | x | Pre-radiation |
| USP9X_41 | 157 | x | x | x |  | x | x |  | x | x | x | x | x |  | x | x | x | Unresolved |
| USP9X_42 | 267 | x | x | x |  | x | x |  | x | x | x | x | x |  | x | x | x | Unresolved |
| USP9X_43 | 96 | x | x | x |  | x | x |  | x | x | x | x | x |  | x | x | x | Unresolved |
| USP9X_44 | 135 | x | x | x |  | x | x |  | x | x | x | x | x |  | x | x | x | Pre-radiation |
| DDX3X_1 | 45 | x | x | x |  | x |  |  | x | x | x | x |  |  | x | x | x | Unresolved |
| DDX3X_2 | 61 | x | x | x |  | x | x |  | x | x | x | x |  |  | x | x | x | Unresolved |
| DDX3X_3 | 48 | x | x | x |  | x | x |  | x | x | x | x | x |  | x | x | x | Unresolved |
| DDX3X_4 | 133 | x | x | x |  | x | x |  | x | x | x | x | x |  | x | x | x | Unresolved |
| DDX3X_5 | 159 | x | x | x |  | x | x |  | x | x | x | x | x |  | x | x | x | Pre-radiation |
| DDX3X_6 | 100 | x | x | x |  | x | x |  | x | x | x | x | x |  | x | x | x | Unresolved |
| DDX3X_7 | 136 | x | x | x |  | x | x |  | x | x | x | x | x |  | x | x | x | Pre-radiation |
| DDX3X_8 | 86 | x | x | x |  | x | x |  | x | x | x | x | x |  | x | x | x | Pre-radiation |
| DDX3X_9 | 99 | x | x | x |  | x | x |  | x | x | x | x | x |  | x | x | x | Unresolved |
| DDX3X_10 | 161 |  | x | x |  | x | x |  | x | x | x | x | x |  | x | x | x | Unresolved |
| DDX3X_11 | 145 |  | x | x |  | x | x |  | x | x | x | x | x |  | x | x | x | Pre-radiation |
| DDX3X_12 | 145 |  | x | x |  | x | x |  | x | x | x | x | x |  | x | x | x | Unresolved |
| DDX3X_13 | 182 | x | x | x |  | x | x |  | x | x | x | x | x |  | x | x | x | Pre-radiation |
| DDX3X_14 | 118 | x | x | x |  | x | x |  | x | x | x | x | x |  | x | x | x | Unresolved |
| DDX3X_15 | 154 | x | x | x |  | x | x |  | x | x | x | x | x |  | x | x | x | Unresolved |
| DDX3X_16 | 149 |  | x | x |  | x | x |  | x | x | x | x | x |  | x | x | x | Unresolved |
| DDX3X_17 | 80 |  | x | x |  | x | x |  | x | x | x | x | x |  | x | x |  | Unresolved |
| UTX_1 | 167 | x | x | x |  | x |  |  | x | x | x | x | x |  |  | x | x | Unresolved |
| UTX_2 | 64 | x | x | x |  | x |  |  | x | x | x | x | x |  | x | x | x | Unresolved |
| UTX_3 | 109 | x | x | x |  | x | x |  | x | x | x | x | x |  | x | x | x | Unresolved |
| UTX_4 | 50 | x | x | x |  | x | x |  | x | x | x | x |  |  |  | x | x | Unresolved |
| UTX_5 | 59 | x | x | x |  | x | x |  | x | x | x | x |  |  | x | x | x | Pre-radiation |
| UTX_6 | 121 | x | x | x |  | x | x |  | x | x | x | x | x |  | x | x | x | Unresolved |
| UTX_7 | 55 | x | x | x |  | x | x |  | x | x | x | x | x |  | x | x | x | Pre-radiation |
| UTX_8 | 35 | x | x | x |  | x | x |  | x | x | x | x | x |  | x | x | x | Unresolved |
| UTX_9 | 94 | x | x | x |  | x | x |  | x | x | x | x | x |  | x | x | x | Pre-radiation |
| UTX_10 | 127 | x | x | x |  | x | x |  | x | x | x | x | x |  | x | x | x | Pre-radiation |
| UTX_11 | 99 | x | x | x |  | x | x |  | x | x | x | x | x |  | x | x | x | Unresolved |
| UTX_12 | 220 | x | x | x |  | x | x |  | x | x | x | x | x |  | x | x | x | Unresolved |
| UTX_13 | 135 | x |  | x |  | x | x |  | x |  | x | x | x |  | x | x | x | No Y coverage |
| UTX_14 | 96 | x | x | x |  | x | x |  | x | x | x | x | x |  | x | x | x | Pre-radiation |
| UTX_15 | 105 | x | x | x |  | x | x |  | x | x | x |  | x |  | x | x | x | Unresolved |
| UTX_16 | 396 | x | x | x |  | x | x |  | x | x | x | x | x |  | x | x | x | Pre-radiation |
| UTX_17 | 785 | x | x | x |  | x | x |  | x | x | x | x | x |  | x | x | x | Pre-radiation |
| UTX_18 | 130 | x | x | x |  | x | x |  | x | x | x | x | x |  | x | x | x | Unresolved |
| UTX_19 | 106 | x | x | x |  | x | x |  | x | x | x | x | x |  | x | x | x | Unresolved |
| UTX_20 | 206 | x | x | x |  | x | x |  | x | x | x | x | x |  | x | x | x | Pre-radiation |
| UTX_21 | 65 | x | x | x |  | x | x |  | x | x | x | x | x |  | x | x | x | Unresolved |
| UTX_22 | 75 | x | x | x |  | x | x |  | x | x | x | x | x |  | x | x | x | Pre-radiation |
| UTX_23 | 149 | x | x | x |  | x | x |  | x | x | x | x | x |  | x |  | x | Pre-radiation |
| UTX_24 | 115 | x | x | x |  | x | x |  | x | x | x | x | x |  | x | x | x | Unresolved |
| UTX_25 | 188 | x | x | x |  | x | x |  | x | x | x | x | x |  | x | x | x | Unresolved |
| UTX_26 | 142 | x | x | x |  | x | x |  | x | x | x | x | x |  | x | x | x | Unresolved |
| UTX_27 | 127 | x | x | x |  | x | x |  | x | x | x | x | x |  | x | x | x | Unresolved |
| UTX_28 | 171 | x | x | x |  | x | x |  | x | x | x | x | x |  | x | x | x | Unresolved |
| UTX_29 | 27 | x | x | x |  | x | x |  | x | x | x | x | x |  | x |  | x | Unresolved |
