# Supplementary material for: Evolution and Survival on Eutherian Sex Chromosomes
Source: PLoS Genet. 2009 Jul 17;5(7):e1000568. doi: 10.1371/journal.pgen.1000568 (PMC2704370; doi:10.1371/journal.pgen.1000568)
Supplement: Table S3 — Comparison of gametolog versus autosomal paralog expression. Expression divergence, measured as the number of tissues out of 11 in which the genes are differentially expressed (see Materials and Methods) is compared for each gametolog pair. X vs. Y represents the number of tissues in which X and Y are differentially expressed, Paralog represents the median number of tissues in which the similarly aged (see Materials and Methods) autosomal paralogs are differentially expressed, and P represents the empirical P value indicating whether the gametolog pair is significantly more differentially expressed than similarly aged autosomal paralogous pairs. (0.05 MB DOC) [file pgen.1000568.s005.doc]

**Table S3. Comparison of gametolog versus autosomal paralog expression**. Expression divergence, measured as the number of tissues out of 11 in which the genes are differentially expressed (see Materials and Methods) is compared for each gametolog pair. X vs. Y represents the number of tissues in which X and Y are differentially expressed, Paralog represents the median number of tissues in which the similarly aged (see Materials and Methods) autosomal paralogs are differentially expressed, and P represents the empirical P value indicating whether the gametolog pair is significantly more differentially expressed than similarly aged autosomal paralogous pairs.

| Gametologs | X vs. Y | Paralog | P |
| --- | --- | --- | --- |
| PRKX/Y | 7 | 7 | 0.441 |
| NLGN4X/Y | 11 | 8 | 0.946 |
| TBL1X/Y | 7 | 7 | 0.455 |
| AMELX/Y | 10 | 8 | 0.733 |
| TMSB4X/Y* | - | - | - |
| CX/Yorf15A | 6 | 7 | 0.333 |
| CX/Yorf15B | 6 | 7 | 0.379 |
| EIF1AX/Y | 11 | 7 | 0.897 |
| ZFX/Y | 11 | 7 | 0.906 |
| USP9X/Y | 9 | 6.5 | 0.661 |
| DDX3X/Y | 6 | 7 | 0.400 |
| UTX/Y | 7 | 7 | 0.441 |

*TMSB4X/Y was not included on the array in [1].

1. Su, A.*I., et a*l. (2004) A gene atlas of the mouse and human protein-encoding transcriptome*s Proc Natl Acad Sci U S* A 101, 6062-6067.
