# Supplementary material for: Evolution and Survival on Eutherian Sex Chromosomes
Source: PLoS Genet. 2009 Jul 17;5(7):e1000568. doi: 10.1371/journal.pgen.1000568 (PMC2704370; doi:10.1371/journal.pgen.1000568)
Supplement: Table S4 — Functional differences between the studied XAR/YAR gametologs. The unique functions reported for either the X copy or the Y copy are listed in each respective column. Functions similar for both the X and the Y copy are listed across both columns. (0.15 MB DOC) [file pgen.1000568.s006.doc]

**Table S4. Functional differences between the studied XAR/YAR gametologs**. The unique functions reported for either the X copy or the Y copy are listed in each respective column. Functions similar for both the X and the Y copy are listed across both columns.

| **Gametologs** | **X copy** | **Y copy** |
| --- | --- | --- |
| **PRKX/Y** | Activates CREB-dependent transcription [1] | PRKX/PRKY translocations are the largest class of infertile males [2] |
| **NLGN4X/Y** | Mutations and deletions lead to variable phenotypes related to intellectual disability [3, 4] |  |
| **TBL1X/Y** | Represses transcription [5] | No repressor activity [6] |
|  |  | Deletion has no phenotypic effect [7] |
| **AMELX/Y** | Mutations cause amelogenesis imperfecta [8] | Deletions have no phenotypic effect [7, 9, 10] |
| **TMSB4X/Y** | Promotes cardiomyocyte migration, survival and repair [11] | Cytotoxic to lymphoblastoid cells [12] |
|  | Participates in coronary vessel development [13] |  |
|  | Induces synthesis of PAI-1 and stimulates PAI-1 binding to endothelial cells [14] |  |
|  | Regulates actin filaments [15] |  |
| **CX/YORF15** | Inhibits ATF4 (to modulate bone mass accrual) [16] |  |
| **EIF1AX/Y** | Involved in ribosome dissociation [17, 18] | Deletion might contribute to azoospermia [19] |
|  | Maintains fidelity of the start codon [20] |  |
| **ZFX/Y** | Regulates stem cell renewal [21] |  |
|  | Activates transcription from the HLA-A11 promoter [22] |  |
| **USP9X/Y** | Participates in chromosome alignment, segregation [23] | Involved in spermatogenesis [24] |
|  | Associates with microtubules in neuronal processes [25] | Has ubiquitin-specific protease activity [26] |
|  | Deubiquitinating enzyme [27] |  |
| **DDX3X/Y** | RNA helicase [28] | Causes sterility in *C. elegans* [29] |
|  | Involved in RNA transcription, RNA splicing, mRNA transport, translation initiation, and cell cycle regulation [30] | Loss results in oligozoospermia or azoospermia in humans [30, 31] |
|  | Participates in HIV and hepatitis C infections, and hepatocellular carcinoma [30] | Fails to overcome a Sxrb-deletion spermatogenic block [32] |
|  | Protein present in meiosis in spermatids [33] | Protein present in spermatogonia [33] |
|  | HIV-1 revRRE export [34] |  |
|  | Candidate tumor suppressor [35] |  |
|  | Shuttles between the nucleus and cytoplasm [31] | |
|  | Rescued a temperature sensitive mutant [31] | |
|  | Translated in many tissues [33] | |
| **UTX/Y** | Binds and activates HOXB1 locus [36] | Encodes HLA-B8-Restricted H-Y antigen [37] |
|  | HIstone H3K27 demethylase [36] | Possible involvement in spermatogenesis [38] |

1. Di Pasquale, G.,Stacey, S. (1998) Adeno-associated virus Rep78 protein interacts with protein kinase A and its homolog PRKX and inhibits CREB-dependent transcriptional activation *J. Virol.* 72, 7916-7925.

2. Jobling, M.*, et al.* (1998) A selective difference between human Y-chromosomal DNA haplotypes *Curr. Biol.* 8, 1391-1394.

3. Chocholska, S.*, et al.* (2006) Molecular cytogenetic analysis of a familial interstitial deletion Xp22.2-22.3 with a highly variable phenotype in female carriers 140, 604-610.

4. Macarov, M.*, et al.* (2007) Deletions of VCX-A and NLGN4: a variable phenotype including normal intellect *J Intellect Disabil Res* 51, 329-333.

5. Yoon, H.-G.*, et al.* (2003) Purification and functional characterization of the human N-CoR complex: the roles of HDAC3, TBL1 and TBLR1 *EMBO J* 22, 1336-1346.

6. Yan, H.-T.*, et al.* (2005) Molecular analysis of TBL1Y, a Y-linked homologue of TBL1X related with X-linked late-onset sensorineural deafness *J Hum Genet* 50, 175-181.

7. Jobling, M.A.*, et al.* (2007) Structural variation on the short arm of the human Y chromosome: recurrent multigene deletions encompassing Amelogenin Y *Hum Mol Genet* 16, 307-316.

8. Wright, J.T. (2006) The molecular etiologies and associated phenotypes of amelogenesis imprefecta *Am J Med Genet Part A* 140A, 2547-2555.

9. Lattanzi, W.*, et al.* (2005) A large interstitial deletion encompassing the amelogenin gene on the short arm of the Y chromosome *Hum Genet* 116, 395-401.

10. Kashyap, V.*, et al.* (2006) Deletions in the Y-derived amelogenin gene fragment in the Indian population *BMC Med Genet* 7, doi: 10.1186/1471-2350-1187-1137.

11. Bock-Marquette, I.*, et al.* (2004) Thymosin beta4 activates integrin-linked kinase and promotes cardiac cell migration, survival and cardiac repair *Nature* 432, 466-472.

12. Smart, N.*, et al.* (2007) Thymosin beta4 induces adult epicardial progenitor mobilization and neovascularization *Nat Genet* 445, 177-182.

13. Clark, E.A.*, et al.* (2000) Genomic analysis of metastasis reveals an essential role for RhoC 406, 532-535.

14. Boncela, J.*, et al.* (2006) Binding of PAI-1 to endothelial cells stimulated by Thymosin B4 and modulation of their fibrinolytic potential *J Biol Chem* 281, 1066-1072.

15. Yu, F.*, et al.* (1994) Effects of thymosin beta 4 and thymosin beta 10 on actin structures in living cells *Cell Motil Cytoskeleton* 27, 13-25.

16. Yu, V.W.*, et al.* (2006) Inhibition of ATF4 transcriptional activity by FIAT/γ-taxilin modulates bone mass accrual *Ann NY Acad Sci* 1068, 131-142.

17. Dever, T.E.*, et al.* (1995) Determination of the amino acid sequence of rabbit, human, and wheat germ protein synthesis factor eIF-4C by cloning and chemical sequencing *J Biol Chem* 269, 3212-3218.

18. Olsen, D.S.*, et al.* (2003) Domains in eIF1A that mediate binding to eIF2, eIF3 and eIF5B and promote ternary complex recruitment in vivo *EMBO J.* 22, 193-204.

19. Kleiman, S.*, et al.* (2007) Expression profile of AZF genes in testicular biopsies of azoospermic men *Hum Reprod* 22, 151-158.

20. Maag, D.*, et al.* (2006) Communication between eukaryotic translation initiation factors 5 and 1A within the ribosomal pre-initiation complex plays a role in start site selection *J Mol Biol* 356, 724-737.

21. Galan-Caridad, J.M.*, et al.* (2007) Zfx controls the self-renewal of embryonic and hematopoietic stem cells *Cell* 129, 345-357.

22. L'Haridon, M.*, et al.* (1996) Transcriptional regulation of the MHC class I HLA-A11 promoter by the zinc finger protein ZFX *Nucleic Acids Res* 24, 1928-1935.

23. Jones, M.H.*, et al.* (1996) The Drosophila developmental gene fat facets has a human homologue in Xp11.4 which escapes X-inactivation and has related sequences on Yq11.2 *Hum Mol Genet* 5, 1695-1701.

24. Brown, G.M.*, et al.* (1997) Characterisation of the coding sequence and fine mapping of the human DFFRY gene and comparative expression analysis and mapping to the Sxrb interval of the mouse Y chromosome of the Dffry gene *Hum Mol Genet* 7, 97-107.

25. Friocourt, G.*, et al.* (2005) Doublecortin interacts with the ubiquitin protease DFFRX, which associates with microtubules in neuronal processes *Mol Cell Neurosci* 28, 153-164.

26. Lee, K.H.*, et al.* (2003) Ubiquitin-specific protease activity of USP9Y, a male infertility gene on the Y *Reprod Fert Dev* 15, 129-133.

27. Chen, X.*, et al.* (2002) A specific protein substrate for a deubiquitinating enzyme: Liquit facets is the substrate of Fat facets *Genes Dev* 16, 289-294.

28. Foresta, C.*, et al.* (2000) Deletion and expression analysis of AZFa genes on the human Y chromosome revealed a major role for DBY in male infertility *Hum Mol Genet* 9, 1161-1169.

29. Chu, D.S.*, et al.* (2006) Sperm chromatin proteomics identifies evolutionarily conserved fertility factors *Nature* 443, 101-105.

30. Rosner, A.,Rinkevich, B. (2007) The DDX3 subfamily of the DEAD box helicase: Divergent roles unveiled by studying different organisms and in vitro assays *Curr Med Chem* 14, 2517-2525.

31. Sekiguchi, T.*, et al.* (2004) Human DDX3XY, the second Y-encoded isoform of RNA helicase DDX3, rescues hamster temperature-sensitive ET24 mutant cell line with a DDX3X mutation. *Exp Cell Res* 300, 213-222.

32. Mazeyrat, S.*, et al.* (2001) A Y-encoded subunit of the translation initiation factor Eif2 is essential for mouse spermatogenesis *Nat Genet* 29, 49-53.

33. Ditton, H.-J.*, et al.* (2004) The AZFa gene DBY (DDX3Y) is widely transcribed but the protein is limited to the male germ cells by translation control *Hum Mol Genet* 13, 2333-2341.

34. Yedavalli, V.S.*, et al.* (2004) Requirement for DDX3 DEAD box RNA helicase for HIV-1 revRRE export function *Cell* 119, 381-392.

35. Chao, C.-H.*, et al.* (2006) DDX3, a DEAD box RNA helicase with tumor growth-suppressive property and transcriptional regulation activity of the p21waf1/cip1 promoter, is a candidate tumore suppresor *Cancer Res* 66, 6579-6588.

36. Agger, K.*, et al.* (2007) UTX and JMJD3 are histone H3K27 demehylases invovled in HOX gene regulation and development *Nature* 449, 731-734.

37. Warren, E.H.*, et al.* (2000) The human UTY gene encodes a novel HLA-B8-restricted H-Y antigen *J Immunol* 164, 2807-2814.

38. Skaletsky, H.*, et al.* (2003) The male-specific region of the human Y chromosome is a mosaic of discrete sequence classes *Nature* 423, 825-837.
