# Supplementary material for: Evolution and Survival on Eutherian Sex Chromosomes
Source: PLoS Genet. 2009 Jul 17;5(7):e1000568. doi: 10.1371/journal.pgen.1000568 (PMC2704370; doi:10.1371/journal.pgen.1000568)
Supplement: Table S5 — Accession numbers for all complete YAR genes, retrieved from GenBank. Listed are the NCBI accession numbers for all available complete coding sequences of orthologous Y-linked genes in mammals, at the time of this study. (0.06 MB DOC) [file pgen.1000568.s007.doc]

**Table S5. Accession numbers for all complete YAR genes, retrieved from GenBank.** Listed are the NCBI accession numbers for all available complete coding sequences of orthologous Y-linked genes in mammals, at the time of this study.

| Gene | NCBI accession | Species |
| --- | --- | --- |
| PRKY | NM_002760 | human |
|  | AY728014 | chimpanzee |
| NLGN4Y | NM_014893 | human |
|  | AY728015 | chimpanzee |
| TBL1Y | NM_033284 | human |
|  | AY958080 | chimpanzee |
| AMELY | NM_001143 | human |
|  | NM_001102459 | chimpanzee |
|  | NM_001085422 | horse |
|  | NM_174240 | cow |
| TMSB4Y | NM_004202 | human |
|  | EF197930, Blat* | chimp |
| CYorf15A | NM_001005852 | human |
|  | NM_001009080 | chimpanzee |
| CYorf15B | NM_032576 | human |
|  | NM_001008989 | chimpanzee |
| EIF1AY | NM_004681 | human |
|  | NM_001008977 | chimpanzee |
| ZFY | NM_003411 | human |
|  | NM_001009003 | chimpanzee |
|  | NM_009571 | mouse |
|  | NM_177491 | cow |
| USP9Y | NM_004654 | human |
|  | NM_001009110 | chimpanzee |
|  | NM_148943 | mouse |
| DDX3Y | NM_001122665 | human |
|  | NM_001008986 | chimpanzee |
|  | NM_012008 | mouse |
| UTY | NM_007125 | human |
|  | NM_001009002, Blat* | chimpanzee |
|  | NM_009484 | mouse |

*Only truncated chimpanzee sequence was available

through NCBI, so complete Y-linked sequence was

extracted from Blat [1] output using chimpanzee X as a query

1. Kent, W.J. (2002) BLAT - The BLAST-like alignment too*l Genome R*es 12, 656-664.
