# Supplementary material for: Evolution and Survival on Eutherian Sex Chromosomes
Source: PLoS Genet. 2009 Jul 17;5(7):e1000568. doi: 10.1371/journal.pgen.1000568 (PMC2704370; doi:10.1371/journal.pgen.1000568)
Supplement: Table S6 — Identification of optimal probe sets. To identify gene-specific probe sets, we used the consensus sequence for each probe set as a query in blastn [1] against the nonreduntant human (A) or mouse (B) genomes. Database hits were considered from known proteins with an e-value less than or equal to 1×10^−20^ and either (1) an identity of 100% and length greater than 49 bp, or (2) an identity higher than 94% and length of at least either 99 bp or 90% of the length of the query. If more than two specific probes were identified, we used the longest one. (0.09 MB DOC) [file pgen.1000568.s008.doc]

**Table S6. Identification of optimal probe sets.** To identify gene-specific probe sets, we used the consensus sequence for each probe set as a query in blastn [1] against the nonreduntant human (A) or mouse (B) genomes. Database hits were considered from known proteins with an e-value less than or equal to 1x10^-20^ and either (1) an identity of 100% and length greater than 49 bp, or (2) an identity higher than 94% and length of at least either 99 bp or 90% of the length of the query. If more than two specific probes were identified, we used the longest one.

A. Human probe sets

| Gene | Probe ID# | # transcripts hit by Blast Search (%id if non-specific) | Specificity |
| --- | --- | --- | --- |
| AMELX | 208410_X_AT | 6 hits; 6X | Specific |
| AMELY | 208220_X_AT | 3 hit; 3Y | Specific |
| CXorf15 | 219969_AT | 2 hits; 2X | Specific |
| CYorf15A | gnf1h00826_at | 1 hit; 1Y | Specific |
| CYorf15B | 214131_AT | 1 hit; 1Y | Specific |
| DDX3X | 201210_AT | 1 hit: 1 X | Specific |
| DDX3Y | 205001_S_AT | 2 hits; 2Y | Specific |
| EIF1AX | 201016_AT | 1 hit; 1X | Specific |
| EIF1AY | 204409_S_AT | 1 hit; 1X | Specific |
| NLGN4X | 221933_AT | 3 hits; 3X | Specific |
| NLGN4Y | 207703_AT | 3 hits; 3Y | Specific |
| PRKX | 204061_AT | 1 hit; 1X | Specific |
| PRKY | 206279_AT | 2 hits; 2Y | Specific |
| TBL1X | 201867_S_AT | 2 hits; 2X | Specific |
| TBL1Y | 211462_S_AT | 3 hits; 3Y | Specific |
| USP9X | 201100_S_AT | 2 hits; 2X | Specific |
| USP9Y | 206624_AT | 1 hit; 1Y | Specific |
| UTX | 203992_S_AT | 6 hits; 6X | Specific |
| UTY | 208067_X_AT | 7 hits; 5Y (100% id), 2X (94% id) |  |
| ZFX | 207920_X_AT | 13 hits; 10X (100% id), 3Y (96% id) |  |
| ZFY | 207246_AT | 8 hits; 3Y (100% id), 5X (95% id) |  |

B. Mouse probe sets

| Gene | Probe ID# | # transcripts hit by Blast Search (%id if non-specific) | Specificity |
| --- | --- | --- | --- |
| AMELX | gnf1m00155_a_at | 2 hits; 2X | Specific |
| AMELY | - | - | - |
| CXorf15 | gnf1m19412_at | 2 hits; 2X | Specific |
| CYorf15 | - | - | - |
| DDX3X | gnf1m02177_s_at | 1 hit: X | Specific |
| DDX3Y | 1426438_at | 3 hits; 3Y | Specific |
| EIF1AX | 201016_AT | 2 hit; 2X | Specific |
| EIF1AY | - | - | - |
| NLGN4X | - | - | - |
| NLGN4Y | - | - | - |
| PRKX | 1424286_at | 2 hit; 2X | Specific |
| PRKY | - | - | - |
| TBL1X | gnf1m17815_a_at | 2 hits; 2X | Specific |
| TBL1Y | - | - | - |
| USP9X | gnf1m13032_s_at | 2 hits; 2X | Specific |
| USP9Y | 1452509_at | 2 hit; 2Y | Specific |
| UTX | gnf1m26304_at | 2 hits; 2X | Specific |
| UTY | 1426598_at | 2 hits; 2Y | Specific |
| ZFX | gnf1m22596_at | 2 hits; 2X | Specific |
| ZFY | 1449978_at | 2 hits; 2Y | Specific |

1. Altschul, S.*F., et a*l. (1997) Gapped BLAST and PSI-BLAST: a new generation of protein database seach program*s Nucleic Acids R*es 25, 3389-3402.
